# Harnessing Sorghum Landraces to Breed High-Yielding, Grain Mold-Tolerant Cultivars With High Protein for Drought-Prone Environments

**DOI:** 10.3389/fpls.2021.659874

**Published:** 2021-06-30

**Authors:** Mallela Venkata Nagesh Kumar, Vittal Ramya, Mahalingam Govindaraj, Chanda Venkata Sameer Kumar, Setaboyine Maheshwaramma, Seshu Gokenpally, Mathyam Prabhakar, Hariprasanna Krishna, Mulinti Sridhar, Maparla Venkata Ramana, Kodari Avil Kumar, Rumandla Jagadeeshwar

**Affiliations:** ^1^Regional Agricultural Research Station, Palem - Professor Jayashankar Telangana State Agricultural University, Hyderabad, India; ^2^Sorghum Improvement Asia Program - International Crop Research Institute for Semi Arid Tropics, Patancheru, India; ^3^Indian Council of Agricultural Research—Central Research Institute for Dryland Agriculture, Hyderabad, India; ^4^Indian Council of Agricultural Research—Indian Institute of Millets Research, Hyderabad, India

**Keywords:** sorghum, landraces, drought tolerance, grain mold, G × E interaction, AMMI, GGE biplot

## Abstract

Intermittent drought and an incidence of grain mold disease are the two major constraints affecting sorghum production and productivity. The study aimed at developing drought-tolerant sorghum varieties possessing a high protein content and tolerance to grain mold with stable performance using additive main effects and multiplicative interaction (AMMI) and genotype and genotype × environment interaction (GGE) biplot methods. Systematic hybridization among the 11 superior landraces resulted in subsequent pedigree-based breeding and selection from 2010 to 2015 evolved 19 promising varieties of grains such as white, yellow, and brown pericarp grains. These grain varieties were evaluated for their adaptability and stability for yield in 13 rainfed environments and for possessing tolerance to grain mold in three hot spot environments. A variety of yellow pericarp sorghum PYPS 2 (3,698 kg/ha; 14.52% protein; 10.70 mg/100 g Fe) possessing tolerance to grain mold was identified as a stable variety by using both AMMI and GGE analyses. Four mega-environments were identified for grain yield and fodder yield. Sorghum varieties PYPS 2, PYPS 4, PYPS 8, and PYPS 11 were highly stable in E2 with a low grain mold incidence. Besides meeting the nutritional demand of smallholder farmers under dryland conditions, these varieties are suitable for enhancing sorghum productivity under the present climate change scenario.

## Introduction

Sorghum [*Sorghum bicolor* (L.) Moench] is a widely adaptable cereal crop cultivated in tropical, subtropical, and temperate regions of the world. It is the fifth most important cereal crop next to wheat, rice, maize, and barley, and it is a staple food for millions of people in the semiarid regions of Asia and Africa (Mundia et al., 2019). Sorghum grew globally in over 40.07 Mha with a production of 57.89 million tons and productivity of 1,444 kg/ha (FAOSTAT, 2019). In India, it is the third most important food grain next to rice and wheat cultivated in an area of 4.09 Mha (FAOSTAT, 2019) in the rainy (*Kharif*) season as a rainfed crop and in the post-rainy (*Rabi*) season under residual soil moisture. More than 90% of the sorghum area and 85% of the production are concentrated in the warm semiarid tracts of central and south India (Davis et al., 2019). The productivity of sorghum in India is still considered to be low at 849 kg/ha compared to the average global productivity of 1,444 kg/ha (FAOSTAT, 2019). This can be attributed to poor soils (marginal lands), unreliable rainfall, incidence of insect pests and diseases, and poor crop input management. The water deficit is significantly increased due to irregularities in rainfall distribution exacerbated by climate change (Eggen et al., 2019; Ocheing et al., 2020). While the rainy season is predominated by the sorghum hybrids, the post-rainy season is dominated by open-pollinated varieties that contribute to low sorghum productivity (Patil et al., 2014). This indicates that a greater emphasis is required for separating and strengthening the focus of rainy and post-rainy breeding on the cultivars' genetic enhancement and their adaptability.

Sorghum has an inherent ability to adapt to a harsh climate. The crop can grow well in dry conditions and can also tolerate water logging, thus making it ideal for cultivation in the arid and semiarid regions of the world (Hadebe et al., 2017). These factors change in relation to climate change, which is predicted to make sorghum production riskier, especially under rainfed agriculture and more so for smallholder farmers (McCarthy and Vlek, 2012). A feasible approach of modifying management practices through a deliberate choice between an improved sorghum variety and local landraces accompanied by an appropriate time of sowing will enhance the adaptive capacity of many resource-poor sorghum farmers and ultimately increase sorghum production ensuring food and livelihood security (Akinseye et al., 2020).

Grain mold is one of the most important diseases in sorghum, which is caused by a complex of fungal species. The genera *Fusarium, Curvularia*, and *Alternaria* are mainly responsible for 80–90% of the infection in India (Das et al., 2020). Species of *Bipolaris, Phoma, Drechslera, Exserohilum, Aspergillus, Cladosporium, Penicillium, Olpitrichum, Gonatobotrytis*, and *Aspergillus* are also detected sporadically in low frequency (Das et al., 2020). The disease can cause yield losses ranging from 30 to 100% depending on the cultivars and weather conditions (Kalaria et al., 2020). Losses in seed weight, grain density, germination, and seed viability due to grain mold lead to a significant decline in seed quality parameters in the rainy season (Nida et al., 2019). Toxins and secondary metabolites produced by the fungi on infected grains render sorghum unfit for human consumption and for cattle and poultry feed (Das et al., 2020). Host-plant resistance is the most cost-effective, efficient, and eco-friendly management practice (Mofokeng et al., 2017). In sorghum, properties such as panicle compactness, glume cover, glume pigmentation, grain hardness, polyphenols (tannins), flavonoids (flavan-4-ols), and antifungal proteins (chitinases, glucanases, sormatin, PR-10, and RIPs) confer a resistance to grain mold disease. There is a need to avoid dependency on a few sources of grain mold-resistant genes and alleles that are currently available, and in this context, crop wild relatives and landraces offer a tremendous scope acting as reservoirs of useful genes for sorghum improvement (Brar and Khush, 2018; Kyratzis et al., 2019). With an increase in the effect of climate change, there is a need to collect, screen, and identify novel sorghum germplasm harboring the grain mold-resistant trait that can be harnessed for adaptation to rainfed agroecologies of India.

Sorghum is a significant source of dietary energy, protein, and micronutrients for the vast majority of the population in sub-Saharan Africa and India (Awika, 2017). It is a good source of phytochemicals including phenolic acid, flavonoids, anthocyanins, phytosterols, plicosanols, tannins, and carotenoids, which make the grain suitable for developing the functional food and nutraceuticals (Balcerek et al., 2020). Additionally, high antioxidant levels in pigmented and tannin sorghum varieties offer many health benefits including slow digestability, cholesterol-lowering, antioxidant, anti-inflammatory, and anticarcinogenic properties (Abdelhalim et al., 2021). The presence of tannins in the testa, which is a layer beneath the pericarp, improves a resistance to grain mold in sorghum (Cuevas et al., 2018). However, red-pigmented testa and high tannin content are less desired in India where red grain sorghum varieties are rarely used for human consumption. Yellow pericarp sorghum is rich in flavanones and has slightly higher total phenolic contents than white sorghum (Dykes et al., 2011). There is a great demand for sorghum with a yellow pericarp owing to high nutritional and good flatbread making and keeping qualities (Jaisimha, 2019). Biofortification of sorghum through genetic approaches and an increased intake of nutrition-rich sorghum grains can help in improving the nutritional security in the developing world (Kumar et al., 2015).

Keeping in view of a narrow genetic diversity for grain minerals in modern sorghum cultivars, the identification and utilization of valuable alleles in wild ancestors of crop plants are considered as a sustainable approach for enhancing sorghum nutrition (Mofokeng et al., 2018; Abdelhalim et al., 2019). India is considered as the secondary center of sorghum diversity next to East Africa (Ananda et al., 2020). High levels of within- and between-population variability among the sorghum landrace collections indicate a high germplasm diversity and a traits-based genetic novelty, which contribute to sorghum yield and adaptation improvement (Ghebru et al., 2002). However, adaptability and stability in yield are often challenged by the presence of a genotype × environment (G × E) interaction, which is a number one factor responsible for changing the genotype performance in different environments. Hence, it is important that multi-environment trials are conducted periodically to investigate the G × E interaction for selecting stable genotypes for yield and other important traits. The additive main effects and multiplicative interaction (AMMI) analysis proposed by Gauch (2013) and the genotype and G × E interaction (GGE) biplot model developed by Yan et al. (2000) are powerful tools used by plant breeders, geneticists, and agronomists for the identification of genotypes with high yield and wide adaptability.

These methods were also used to identify the landraces with yield stability and adaptability in sorghum (Admas and Tesfaye, 2017), chickpea (Pouresmael et al., 2018), wheat (Bavandpori et al., 2018), common bean (Philipo et al., 2020), etc. for further use in breeding programs for the development of new varieties. In sorghum, the high genetic and phenotypic diversity were reported from the landraces collected from India (Elangovan et al., 2009, 2012; Vara Prasad and Sridhar, 2019), Ethiopia (Adugna, 2014; Amelework et al., 2015; Derese et al., 2018; Wondimu et al., 2020), Eritrea (Tesfamichael et al., 2014), and Sudan (Abdelhalim et al., 2021). These landraces are the indispensable sources of genetic variation that can be utilized by plant breeders in the development of improved varieties with higher productivity, nutrients, grain mold tolerance, and climate resilience (Dwivedi et al., 2016; Godwin et al., 2019).

An attempt was made in this study to utilize the sources of variation for grain mold tolerance and a protein content present in sorghum landraces, which were evolved under vulnerable conditions with low inputs after the continuous selection by the farmers. To our knowledge, this is the first study to identify stable, high-yielding, drought- and grain mold-tolerant, and nutritionally rich sorghum varieties that were developed from the landraces in the Indian subcontinent. The objectives of the study were to (1) collect the landraces from the southern and central parts of India and identify agronomically superior landraces with a resistance to grain mold disease; (2) develop potential sorghum varieties from the superior landraces; (3) identify high-yielding, grain mold-tolerant genotypes with a stable performance using AMMI and GGE analyses; and (4) determine the nutrient composition [starch, sugar, protein, iron (Fe), and zinc (Zn)] of the developed sorghum varieties.

## Materials and Methods

### Collection and Maintenance of Landraces

A total of 108 landraces were collected from various locations in the southern and central parts of India in 2008. These landraces represented a diversity for grain maturity, grain color, panicle shape, grain yield, fodder yield, porridge making quality, fodder quality, and tolerance to grain mold disease under field conditions (Supplementary Table 1). In the field evaluation of these landraces from 2008 to 2010, a single-plant selection was followed by self-pollinating main panicles of individual landrace collections for three generations to bring a genetic uniformity within the landraces at the Regional Agricultural Research Station (RARS), Palem, Telangana (Former Andhra Pradesh), India. Based on the construction of passport data and dendrogram (Supplementary Figures 1, 2) using the distances matrix obtained by an unweighted pair group method with arithmetic mean (UPGMA), 36 diverse landraces (PSLRC 1–PSLRC 36) distinct for various characters, *viz.*, maturity, grain type, tolerances to grain mold disease, and terminal moisture stress, were maintained for future breeding (Table 1).

**Table 1:** Description of the superior sorghum landraces used in this study.

**S. No**.	**Local accession Number**	**Sorghum race**	**Days to 50% flowering**	**Plant Height (cm)**	**Glume cover-age %**	**Grain color**	**Panicle compactness**	**Grain mold resistance**
1	PSLRC 1	Durra	65	285	71	Yellow	Semi compact	Tolerant
2	PSLRC 2	Durra	68	315	83	Yellow	Semi compact	Tolerant
3	PSLRC 3	Guinea	68	246	79	White	Semi compact	Tolerant
4	PSLRC 4	Durra	66	220	67	Yellow	Semi compact	Tolerant
5	PSLRC 5	Guinea	71	264	54	White	Compact	Susceptible
6	PSLRC 6	Durra	69	310	63	Yellow	Semi compact	Tolerant
7	PSLRC 7	Durra	68	240	71	Yellow	Semi compact	Tolerant
8	PSLRC 8	Durra	65	298	90	Brown	Semi compact	Tolerant
9	PSLRC 9	Durra	71	210	85	Brown	Semi compact	Tolerant
10	PSLRC 10	Durra	72	218	46	Brown	Semi compact	Tolerant
11	PSLRC 11	Durra	69	195	62	Brown	Semi compact	Susceptible
12	PSLRC 12	Durra	68	226	76	Yellow	Semi compact	Tolerant
13	PSLRC 13	Durra	65	315	88	Yellow	Semi compact	Tolerant
14	PSLRC 14	Durra	62	242	77	Yellow	Semi compact	Tolerant
15	PSLRC 15	Durra	64	292	90	Brown	Semi compact	Tolerant
16	PSLRC 16	Durra	66	262	72	Yellow	Semi compact	Tolerant
17	PSLRC 17	Durra	65	245	75	Brown	Semi compact	Tolerant
18	PSLRC 18	Durra	64	272	80	Yellow	Semi compact	Tolerant
19	PSLRC 19	Durra	71	282	69	Yellow	Compact	Tolerant
20	PSLRC 20	Guinea	78	265	54	Black glume	Loose	Tolerant
21	PSLRC 21	Guinea	76	272	78	Black glume	Loose	Tolerant
22	PSLRC 22	Guinea	64	218	43	Black glume	Loose	Tolerant
23	PSLRC 23	Durra	75	234	68	Brown	Semi compact	Tolerant
24	PSLRC 24	Durra	72	240	75	Brown	Semi compact	Tolerant
25	PSLRC 25	Durra	76	265	56	Brown	Loose	Tolerant
26	PSLRC 26	Durra	75	245	70	Yellow	Semi compact	Tolerant
27	PSLRC 27	Durra	68	262	38	Brown	Compact	Tolerant
28	PSLRC 28	Durra	62	275	81	Yellow	Semi compact	Tolerant
29	PSLRC 29	Guinea	65	260	75	White	Compact	Tolerant
30	PSLRC 30	Guinea	66	235	68	White	Semi compact	Tolerant
31	PSLRC 31	Durra	72	292	91	Brown	Loose	Tolerant
32	PSLRC 32	Durra	70	210	94	Yellow	Semi compact	Tolerant
33	PSLRC 33	Durra	68	228	79	Yellow	Semi compact	Tolerant
34	PSLRC 34	Durra	65	235	82	Yellow	Semi compact	Tolerant
35	PSLRC 35	Durra	65	242	69	Yellow	Semi compact	Tolerant
36	PSLRC 36	Durra	65	262	78	Yellow	Semi compact	Tolerant

**Figure 1:**
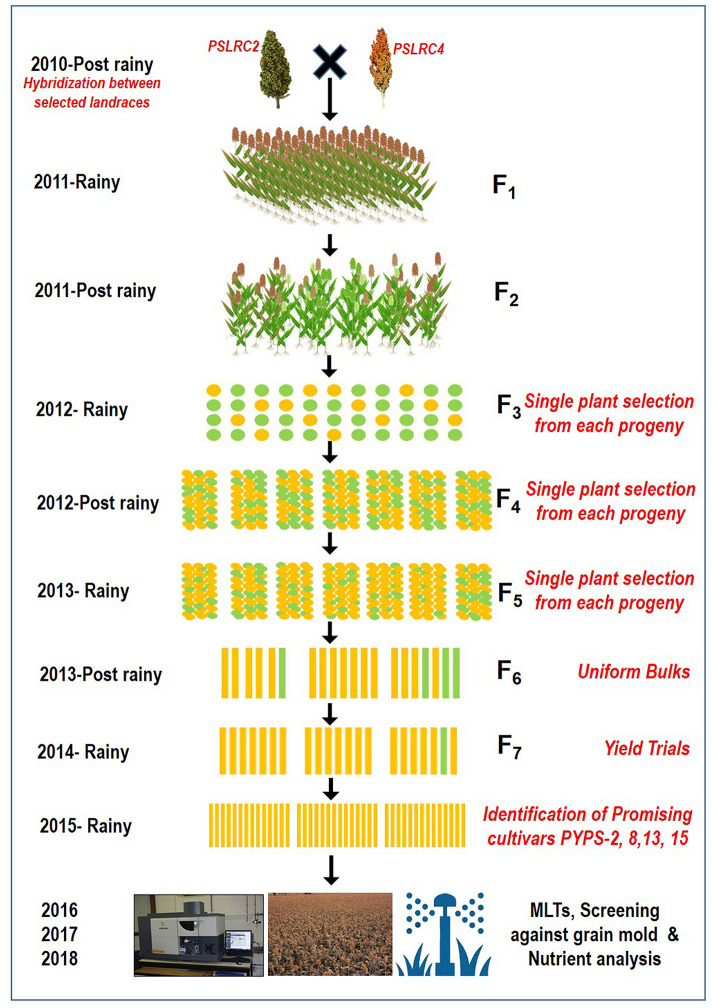
A flow diagram showing the development of sorghum varieties utilizing landraces through a pedigree selection (2010–2015) followed by multi-environment trials (2016–2018).

*The above landraces were selected to develop varieties after diversity analysis of 108 landraces following an unweighted pair group method with arithmetic mean (UPGMA) (Supplementary Table 1 and Figure 1)*.

### Development of Sorghum Varieties

From 2010 to 2015, hybridization followed by a selection was carried out in rainy and post-rainy seasons by utilizing 11 agronomically superior and grain mold-resistant landraces. A minimum population of 250 plants was maintained in each F_2_ and subsequent generation. They were advanced to F_6_ generation by using the pedigree method of selection. The F_6_ progeny of individual cross combination was considered as a single-sorghum advanced genotype having a diverse genetic background for agronomic and grain characters and grain mold resistance (Figure 1). About 19 superior advanced sorghum cultivars were identified after the evaluation for two consecutive seasons (rainy and post-rainy seasons, 2014) in an advanced variety trial at RARS, Palem, Telangana, India, among which seven genotypes were characterized with a yellow pericarp and six genotypes were with a brown pericarp. The remaining six genotypes had a white grain and a white grain with black glume (Table 2, Supplementary Figures 3, 4).

**Table 2:** Sorghum genotypes evolved through hybridization followed by a selection from the superior landraces.

**S. No**.	**Genotype**	**Cross combination**	**Grain color**
1.	PYPS-1	PSLRC 2 × PSLRC 3	Yellow
2.	PYPS-2	PSLRC 2 × PSLRC 4	Yellow
3.	PYPS-3	PSLRC 3 × PSLRC 4	White
4.	PYPS-4	PSLRC 2 × PSLRC 6	Yellow
5.	PYPS-5	PSLRC 21 × PSLRC 7	White grain with black glume
6.	PYPS-6	PSLRC 3 × PSLRC 6	White
7.	PYPS-7	PSLRC 3 × PSLRC 7	White
8.	PYPS-8	PSLRC 8 × PSLRC 9	Brown
9.	PYPS-9	PSLRC 8 × PSLRC 10	Brown
10.	PYPS-10	PSLRC 9 × PSLRC 10	Brown
11.	PYPS-11	PSLRC 4 × PSLRC 6	Yellow
12.	PYPS-12	PSLRC 4 × PSLRC 12	Yellow
13.	PYPS-13	PSLRC 2 × PSLRC 21	Yellow
14.	PYPS-14	PSLRC 20 × PSLRC 21	White grain with black glume
15.	PYPS-15	PSLRC 2 × PSLRC 7	Yellow
16.	PYPS-16	PSLRC 20 × PSLRC 7	White grain with black glume
17.	PYPS-17	PSLRC 2 × PSLRC 8	Brown
18.	PYPS-18	PSLRC 3 × PSLRC 8	Brown
19.	PYPS-19	PSLRC 4 × PSLRC 8	Brown

### Nutritional Composition Analysis

The nutritional composition analysis of 19 sorghum varieties evaluated at E1 (Palem) in 2018 was performed at MFPI-Quality Control Laboratory, Prof. Jayashankar Telangana State Agricultural University, Hyderabad, Telangana, India. Whole grains were collected from the fields where they were grown and analyzed for the protein, total starch, sugar, Fe, and Zn content. The protein level was quantified by using the generic combustion method of analysis with the LECO F-528 nitrogen analyzer (LECO, St. Joseph, MI, USA) calibrated with ethylenediaminetetraacetic acid (EDTA) according to the association of official analytical chemists method (AOAC, 2016). The grain samples were ground to a suitable fineness to pass No. 20 sieve and dried at 102 ± 2°C for 2 h. A moisture-free sample weighing 200 mg was analyzed to estimate protein content. Analyses for total starch, sugar, Fe, and Zn were performed according to Shegro et al. (2012). Starch content was determined by using a total starch assay procedure. The total sugar content in stalks at physiological maturity was estimated as the total soluble sugars by using a handheld refractometer. For the determination of Fe and Zn contents, sorghum grains were ground to a fine powder. About 2-g flour samples were oven-dried for 3 h after which the samples were triple acid digested by the addition of 1 ml of 55% (v/v) HNO_3_ after cooling. The acid was evaporated by using a sand bath and the samples were oven-dried again. The samples were moistened by using 10 ml of 55% HNO_3_ (1:2 v/v), and they were then placed in the sand bath for 5–10 min. The samples were allowed to dissolve overnight to extract the minerals accordingly.

### Evaluation of Varieties for Yield Performance and Screening for Grain Mold Tolerance

About 19 advanced sorghum varieties along with a popular variety (CSV 31) were evaluated over the three rainy seasons (June–September) from 2016 to 2018 at 13 locations for yield and yield-related characters (Table 3). A single location tested for three consecutive seasons of 2016, 2017, and 2018 was considered as one environment. All 13 environments are drought-prone environments characterized by intermittent dry spells with E4, E6, E8, E9, and E11 receiving an annual rainfall of ≤ 600 mm (Table 3). Each sorghum genotype was planted on six rows of 5-m length plot by using between- and within-row spacing of 45 and 10 cm, respectively. Weeds, insect pests, and foliar disease control were carried out as recommended for the crop by using a combination of chemical and cultural practices. During harvest, the four central rows within each plot were sampled for grain yield and fodder yield. The 19 genotypes along with susceptible (SPV 462) and resistant (IS 8545) checks were also evaluated in the sorghum grain mold nursery over the three rainy seasons (June–September) in 2016 to 2018 at three locations *viz.*, RARS, Palem, Agricultural Research Station, Tandur, Agricultural Research Station, Madhira, Telangana, India under natural epiphytotic conditions for a grain mold evaluation. Each genotype was sown in six rows of 5 m in length during the first fortnight of June so that the grain maturity stage coincided with the periods of frequent rainfall received in the ensuing August–September, thus predisposing the crop to grain mold disease. During rain-free days, high relative humidity (>90%) was maintained from the flowering to the physiological maturity stage by using sprinkler irrigation. About 10 uniformly flowering plants with the same flowering window were tagged in each row. The visual panicle grain mold rating (PGMR) was taken on each of the tagged plants at the prescribed physiological maturity by using a progressive 1–9 scale, where 1 = no mold infection, 2 = 1–5%, 3 = 6–10%, 4 = 11–20%, 5 = 21–30%, 6 = 31–40%, 7 = 41–50%, 8 = 51–75%, and 9 = 76–100% molded grains on a panicle (Singh and Bandyopadhyay, 2000; Thakur et al., 2007). All the trials at each location were conducted in a complete randomized block design with three replications.

**Table 3:** Details of the 13 environments tested for yield and stability of 19 sorghum genotypes in Telangana, India.

**Environment**	**Location**	**Latitude**	**Longitude**	**Soil type**	**Rainfall (mm)**
E1	Palem	16.5461° N	78.2077° E	Red sandy	690
E2	Tandur	17.2576° N	77.5875° E	Sandy loam	780
E3	Madhira	16.9182° N	80.3633° E	Sandy loam	750
E4	Hanwada, Mahabubnagar	16.8106° N	77.9196° E	Red sandy	600
E5	Kodangal	17.1103° N	77.6235° E	Sandy loam	760
E6	Gaddamallaihguda	17.0974° N	78.6867° E	Red sandy	560
E7	PA Pally	16.6996° N	79.0267° E	Sandy loam	700
E8	Maddur	15.8563 N	77.2431° E	Sandy loam	600
E9	Aler	17.6437° N	79.0430° E	Red sandy	580
E10	Kulkacherla	17.0161° N	77.8746° E	Red sandy	630
E11	Ramapuram	15.9653° N	77.9410° E	Red sandy	580
E12	Kothakota	16.3787° N	77.9410° E	Red sandy	720
E13	Devarakadara	16.6248° N	77.8410° E	Red sandy	650

### Data Analysis

Combined ANOVA was performed for yield and disease reaction at 13 and 3, environments, respectively. Statistical computations and estimations were carried out by using GenStat 18.0 (Goedhart and Thissen, 2010) to partition the yield variation into environments, GGE. The grain yield, fodder yield, and disease resistance reaction data were subjected to the AMMI and GGE biplot analysis. The AMMI model combines both additive and multiplicative components of two-way data structures, which helps in the prediction of potential genotypes and an environmental effect on them (Gauch and Zobel, 1996; Gauch, 2013). The GGE biplots were constituted from the first two principal components (PC1 and PC2) derived by subjecting the environment-centered yield data (which contains GGE) to singular-value decomposition (SVD). The model for a GGE biplot (Yan et al., 2000) based on SVD of the first two principal components (PC) is:

$$\begin{array}{l} Y_{ij}=\mu +\beta_{j}+\sum_{\text{k}=1}^{\text{k}}\lambda_{k}\gamma_{ik}\;\delta_{jk} \end{array}$$

where *Y*_*ij*_ is the mean of genotype *i* in environment *j*; μ is the grand mean; β_*j*_ is the environment main effect; *k* is the number of PC required for appropriate depiction of GGE, *n* is the singular value; λ_*k*_ is the singular value of the *k*th PC (PC_*k*_). γ_*ik*_ and δ_*jk*_ are the scores of *i*th genotype and *j*th environment, respectively, for PC_*k*_.

The GGE biplot software was used to generate graphs showing (1) a “which-won-where” pattern to identify mega-environments, (2) ranking of varieties based on yield and stability, and (3) a correlation of vectors between the environments as per the method described by Yan and Kang (2003).

## Results

### AMMI Analysis

The combined ANOVA analysis showed highly significant (*p* ≤ 0.05) genotype differences over locations and seasons suggesting that both grain and fodder yields varied across the environments. Highly significant environments, genotypes, and G × E interaction explained 35.3, 23.6, and 29.8% of the total sum of squares for grain yield and 28.9, 23.4, and 25.0% for fodder yield, respectively (Table 4). The magnitude of the environments (E) and G × E interaction sum of squares were twice larger than that for genotypes sum of squares indicating ample of variations in the genotypic response across the environments for both grain yield (58.9%) and fodder yield (53.9%). Further partitioning of the G × E interaction sum of squares resulted in two significantly interaction PC axes (IPCA1 and IPCA2), which explained 35.9 and 20.7% of the variation, respectively, and together contributed to 56.6% of the total G × E interaction for grain yield. Similarly, for fodder yield, IPCA1 and IPCA2 explained 54.9 and 11.9% of the G × E interaction, respectively, and together contributed to 66.8% of the total variation. This explained the differential performance of genotypes for grain yield and fodder yield across the environments.

**Table 4:** Additive main effects and multiplicative interaction (AMMI) ANOVA for grain yield and fodder yield of 19 sorghum genotypes evolved from landraces over 13 locations in 3 years (2016–2018).

	**Grain Yield**					**Fodder Yield**			
**Source**	**DF**	**SS**	**MS**	**F**	**% contribution SS**	**SS**	**MS**	**F**	**% contribution SS**
Total	2,222	1,087,099,555	489,244	–		36,509,998,551	16431142.46		
Genotypes	18	324,366,092	18,020,338^**^	520.53	23.6	8,547,745,363	474874742.38^**^	200.98	23.4
Environments	12	384,294,081	32,024,506^**^	925.05	35.3	10,581,124,368	881,760,364^**^	373.20	28.9
Blocks	26	53,982,701	2,076,257	59.97	4.96	2,582,926,484	99343326.30	420.85	7.07
Interaction	216	256,949,082	1,189,580^**^	34.4	29.8	9,127,499,637	42256942.76^**^	17.88	25.0
IPCA1	29	92244720.43	3180852.42	91.88	35.9	5010997300.71	172793010.36	73.13	54.9
IPCA2	27	53,188,460	1,969,943	56.90	20.7	1086172456.80	40228609.51	170.24	11.9
Residuals	160	111515901.6	96,356	3221.23	43.7	3,030,329,880	18,939,562	1282.58	33.2
Error	1,950	67,507,598	34,619	–		4,607,225,870	2362679.93		

** *significant at 1% probability level*.

*DF, Degrees of freedom; SS, Sum of squares; MS, Mean sum of squares; F, F-calculated value; IPCA, Interaction principal component axis*.

### AMMI Stability Value

The AMMI stability value (ASV) proposed by Purchase et al. (2000) is used to identify stable genotypes and environments. For environments and genotypes, a low ASV indicates that the environments and genotypes are highly stable and least interactive whereas a high ASV indicates that the environments and genotypes are highly interactive and unstable. Based on the ASV for grain yield, E5, E11, and E13 were the most stable and high yielding environments (Table 5). On the contrary, E4 followed by E10 was the most unstable and most interactive environment with high ASV scores for grain yield. For fodder yield, the environments E6 and E10 were mostly stable with low ASV scores, and the environments E5, E1, and E4 were mostly unstable with high ASV scores (Table 5).

**Table 5:** Mean performance for grain yield, fodder yield, IPCA1, IPCA2 scores, and ASV values of 13 environments.

**S. No**.	**Environment**	**Grain yield (kg/ha)**	**IPCA1**	**IPCA2**	**ASV**	**Fodder yield (kg/ha)**	**IPCA1**	**IPCA2**	**ASV**
1	E1	3,398	−10.36	−1.58	17.96	15,970	−41.17	1.82	189.00
2	E2	2,860	−8.68	−6.32	16.27	13,893	−20.58	−2.80	94.30
3	E3	2,707	2.90	16.71	17.40	12,707	34.07	5.56	156.80
4	E4	2,417	18.31	−10.83	33.40	11,705	37.63	−23.84	174.60
5	E5	3,396	1.85	2.54	4.06	15,968	−43.31	−5.13	199.06
6	E6	2,883	−11.63	−5.58	16.00	13,878	0.88	−15.41	15.90
7	E7	2,716	0.57	13.30	13.30	12,776	14.04	17.09	66.70
8	E8	2,699	10.02	0.01	17.32	12,931	16.29	−8.10	75.20
9	E9	3,076	6.07	−11.80	15.80	14,591	−8.47	−31.76	50.20
10	E10	3,044	−18.98	−4.60	32.30	14,314	−5.27	7.79	25.40
11	E11	2,865	−2.97	7.46	9.00	13,844	−17.66	17.40	82.84
12	E12	2,659	6.72	6.79	13.40	12,852	21.44	3.69	98.50
13	E13	2,725	6.20	−6.10	12.30	12,996	12.10	33.71	57.00
	Overall mean	2880.38				13725.10			
	LSD (0.05)	341.45				1159.50			

*IPCA, Interaction principal component axis; ASV, AMMI stability value; LSD, Least significant difference*.

Sorghum varieties PYPS 2, PYPS 5, PYPS 8, PYPS 13, PYPS 14, and PYPS 17 were the most stable varieties for grain yield, and PYPS 11, PYPS 16, PYPS 7, PYPS 4, and PYPS 18 were the most unstable varieties (Table 6). For fodder yield, the varieties PYPS 16, PYPS 10, PYPS 13, PYPS 15, and PYPS 2 were the most stable, and PYPS 8, PYPS 5, and PYPS 9 were unstable (Table 6).

**Table 6:** Classification of stable sorghum varieties based on mean performance, ASV and stability index for grain yield, fodder yield, disease reaction, and protein.

**S. No**.	**Genotype**	**Grain yield (kg/ha) and its rank**	**IPCA1**	**IPCA2**	**ASV and RASV**	**GSI and RGSI**	**Fodder yield (kg/ha) and its rank**	**IPCA1**	**IPCA2**	**ASV and RASV**	**GSI and RGSI**	**Disease Score and rank**	**ASV and RASV**	**GSI and RGSI**	**Protein (%) and rank**
1	PYPS-1	2,756 (7)	−0.36	5.56	5.6 (7)	13 (4)	14,108 (6)	−7.79	−1.13	35.8 (9)	14 (5)	4.52 (10)	0.01 (1)	11 (2)	12.32 (4)
2	PYPS-2	3,698 (1)	−2.46	0.96	4.4 (3)	4 (1)	20,586 (1)	−23.99	20.70	18.6 (7)	8 (2)	3.92 (3)	0.6 (18)	21 (14)	14.52 (1)
3	PYPS-3	2,639 (14)	−6.14	−5.09	11.7 (12)	25 (13)	12,448 (12)	7.95	1.06	36.5 (10)	21 (10)	4.5 (8)	0.11 (10)	18 (10)	9.78 (15)
4	PYPS-4	2,603 (18)	−12.54	2.18	21.8 (16)	33 (18)	13,008 (9)	20.84	6.84	96 (16)	24 (13)	4.1 (5)	0.4 (16)	21 (15)	10.3 (14)
5	PYPS-5	2,514 (20)	−1.09	−1.00	2.1 (1)	20 (9)	11,793 (16)	26.90	16.18	124.7 (18)	33 (19)	4.51 (9)	0.09 (7)	16 (7)	10.52 (12)
6	PYPS-6	2,604 (17)	−2.26	8.07	8.9 (8)	24 (12)	12,335 (13)	−9.22	−14.77	44.8 (12)	24 (13)	4.73 (13)	0.32 (13)	26 (16)	9.63 (16)
7	PYPS-7	2,575 (19)	11.81	13.00	24.2 (17)	35 (19)	13,059 (8)	7.76	−12.63	37.8 (11)	18 (7)	4.18 (6)	0.18 (11)	17 (8)	9.25 (18)
8	PYPS-8	3,584 (2)	−0.74	−2.43	2.7 (2)	4 (1)	18,632 (2)	−49.35	15.59	227.5 (19)	21 (10)	3.71 (2)	0.04 (3)	5 (1)	13.26 (3)
9	PYPS-9	2,712 (12)	2.64	8.65	9.8 (9)	20 (9)	11,984 (15)	22.22	−12.69	102.8 (17)	31 (17)	4.78 (15)	0.09 (7)	22 (15)	14.13 (2)
10	PYPS-10	2,619 (16)	−0.32	9.98	10.0 (10)	25 (13)	11,538 (18)	2.22	2.37	10.81 (2)	20 (8)	5.1 (18)	0.37 (15)	33 (19)	9.58 (17)
11	PYPS-11	2,699 (13)	−18.36	1.28	31.8 (19)	31 (17)	13,895 (7)	9.28	26.82	50.3 (14)	20 (8)	3.65 (1)	0.23 (12)	13 (5)	10.73 (8)
12	PYPS-12	2,729 (9)	−6.61	−2.17	11.6 (11)	18 (7)	12,728 (10)	0.83	−15.84	16.2 (5)	14 (5)	4.8 (16)	0.06 (4)	20 (12)	10.95 (6)
13	PYPS-13	3,514 (3)	0.88	−4.65	4.6 (4)	7 (3)	18,122 (3)	−2.55	−2.00	11.8 (3)	6 (1)	3.97 (4)	0.09 (7)	11 (4)	10.66 (10)
14	PYPS-14	2,718 (10)	1.90	−3.31	4.7 (5)	14 (6)	11,392 (20)	3.13	8.36	16.6 (6)	25 (15)	5.32 (19)	0.07 (5)	14 (6)	10.52 (12)
15	PYPS-15	3,432 (4)	−3.74	−14.39	14.4 (14)	18 (7)	16,955 (4)	−0.20	−12.80	12.8 (4)	8 (2)	4.54 (11)	0.08 (6)	17 (9)	8.78 (19)
16	PYPS-16	3,268 (5)	17.02	−10.70	31.3 (18)	23 (11)	12,561 (11)	1.01	0.98	4.74 (1)	11 (4)	5 (17)	0.02 (2)	19 (11)	10.77 (7)
17	PYPS-17	2,729 (8)	2.80	1.21	4.9 (6)	13 (4)	11,697 (17)	16.07	14.06	75.3 (15)	31 (17)	4.74 (14)	0.5 (17)	31 (18)	11.28 (5)
18	PYPS-18	2,713 (11)	11.86	−3.92	20.8 (15)	25 (13)	11,627 (19)	9.62	−13.32	46.15 (13)	30 (16)	4.73 (12)	0.72 (19)	31 (17)	10.6 (11)
19	PYPS-19	2,620 (15)	6.98	3.78	12.6 (13)	27 (16)	12,309 (14)	4.23	20.70	27.9 (8)	21 (10)	4.39 (7)	0.32 (13)	20 (13)	10.72 (9)
	CSV 31	2,896 (6)		−3.26			16,588 (5)	−1.62	−11.36						
	Grand mean	2881.1					13868.3								
	LSD (0.05)	412.68					2889.87					2.54			2.15

*Value in the parenthesis indicates rank*.

*IPCA, Interaction principal component axis; ASV, AMMI stability value; RASV, Rank of ASV; GSI, Genotype stability index; RGSI, Rank of GSI; LSD, Least significant difference*.

### Genotype Stability Index

Genotype stability index (GSI) can be used to classify stable genotypes incorporating both yield and stability in a single non-parametric index (Singh et al., 2019). The GSI considered the ranks of the genotype yield across the environments and ASVs. GSI was calculated as the sum of Rank of ASV [RASV (ASV)] and RY (Rank of mean genotype yield of all environments). Considering high grain and fodder yields, a high protein content and moderate resistance to grain mold, low ASV values and high GSI, sorghum varieties PYPS 1 and PYPS 13 were identified as the best stable genotypes. Sorghum varieties PYPS 2, PYPS 8, PYPS 12, PYPS 15, and PYPS 16 having high grain and fodder yields with either a low ASV or a high GSI were also known as the best stable varieties across the environments (Table 6).

### AMMI Biplot Analysis

**Figure 2:**
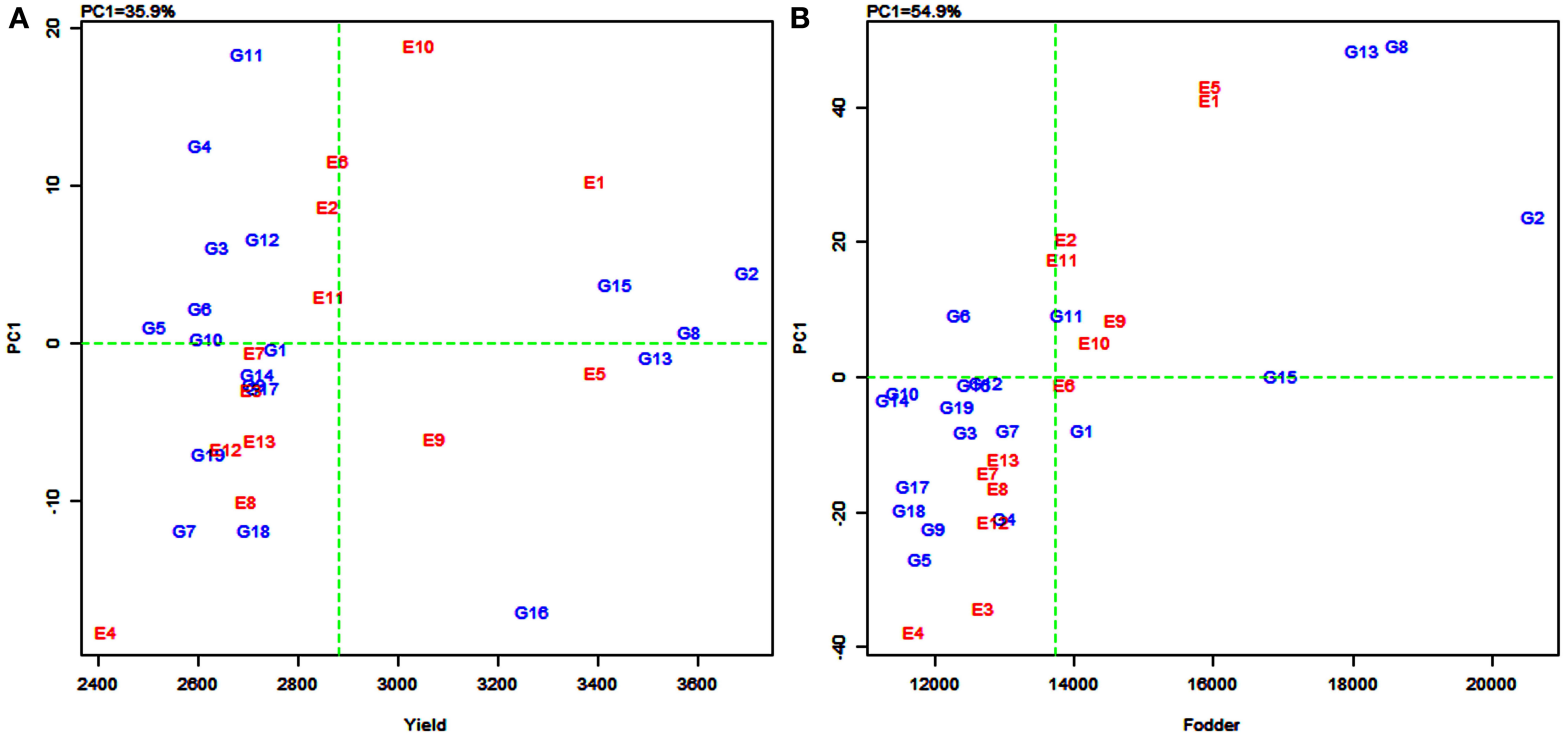
Additive main effects and multiplicative interaction (AMMI1) biplot depicting additive effects vs. interaction principal component axes (IPCA1) for grain yield **(A)** and fodder yield **(B)** of 19 sorghum varieties tested across 13 environments.

In the AMMI1 and AMMI2 biplots (Figure 2), the environments were designated by the letter “E” followed by numbers 1–13 as suffix while the genotypes were represented by the letter “G” followed by numbers 1–19. In the AMMI1 biplot, the main effects (genotype mean and environment mean) on abscissa were plotted against the respective IPCA1 scores on the ordinate (Yan et al., 2007). The quadrants (Q) in the AMMI1 graph represent: higher mean (QI and QII), lower mean (QIII and QIV), +ve IPCA1 score (QI and QIV), and –ve IPCA1 score (QII and QIII). When a genotype and an environment have the same sign on the IPCA1 axis, their interaction is positive and, if opposite, their interaction is negative. Thus, if a variety has a IPCA1 score nearer to zero, it has a small interaction effect and was considered as stable over wide environments. On the other hand, genotypes with high mean yield and large IPCA1 scores were considered as explicitly adapted to specific environments (Abdi and Williams, 2010).

Accordingly, in the present study, sorghum varieties PYPS 2, PYPS 8, and PYPS 15 were specifically adapted to the high-yielding environments E1 and E10 and the varieties PYPS 13 and PYPS 16 were adapted to the environments E5 and E9 with grain yield more than the grand average yield (Figure 2A). Furthermore, the varieties PYPS 2, PYPS 8, and PYPS 13 were also more stable in comparison to PYPS 4, PYPS 7, PYPS 11, and PYPS 16. Similarly, the varieties PYPS 1, PYPS 5, PYPS 6, PYPS 10, and PYPS 14 were nearer to zero indicating that they are highly stable for grain yield than other varieties. For fodder yield, the varieties PYPS 2, PYPS 13, and PYPS 8 were specifically adapted to high-yielding environments E1, E5, E9, and E10 (Figure 2B). The varieties PYPS 10, PYPS 12, PYPS 15, and PYPS 16 were more stable in comparison to PYPS 4, PYPS 5, PYPS 9, PYPS 17, and PYPS 8 as these genotypes were far from the origin. The varieties PYPS 11, PYPS 15, and PYPS 16 were nearer to zero indicating a higher stability for fodder yield than the other genotypes.

The AMMI2 biplot is a graphical representation of the interaction effect wherein the relationship between the genotypes and environments is depicted in a vector view (Guerra et al., 2009). The biplot detects the environments and genotypes that contributed least to the interaction (most stable) as well as the desirable combinations of genotypes and environments in terms of specific adaptability. The statistically stable genotypes and environments are represented by the points nearer to the origin in the AMMI2 biplot, with the values being nearer to zero for the two axes of interaction (IPCA1 and IPCA2). The discrimination power of a test environment is proportional to the length of the environment vector, which is the line connecting the origin and test environment and those genotypes falling apart from the origin with long spokes were termed as highly interacting genotypes (Yan and Holland, 2010).

**Figure 3:**
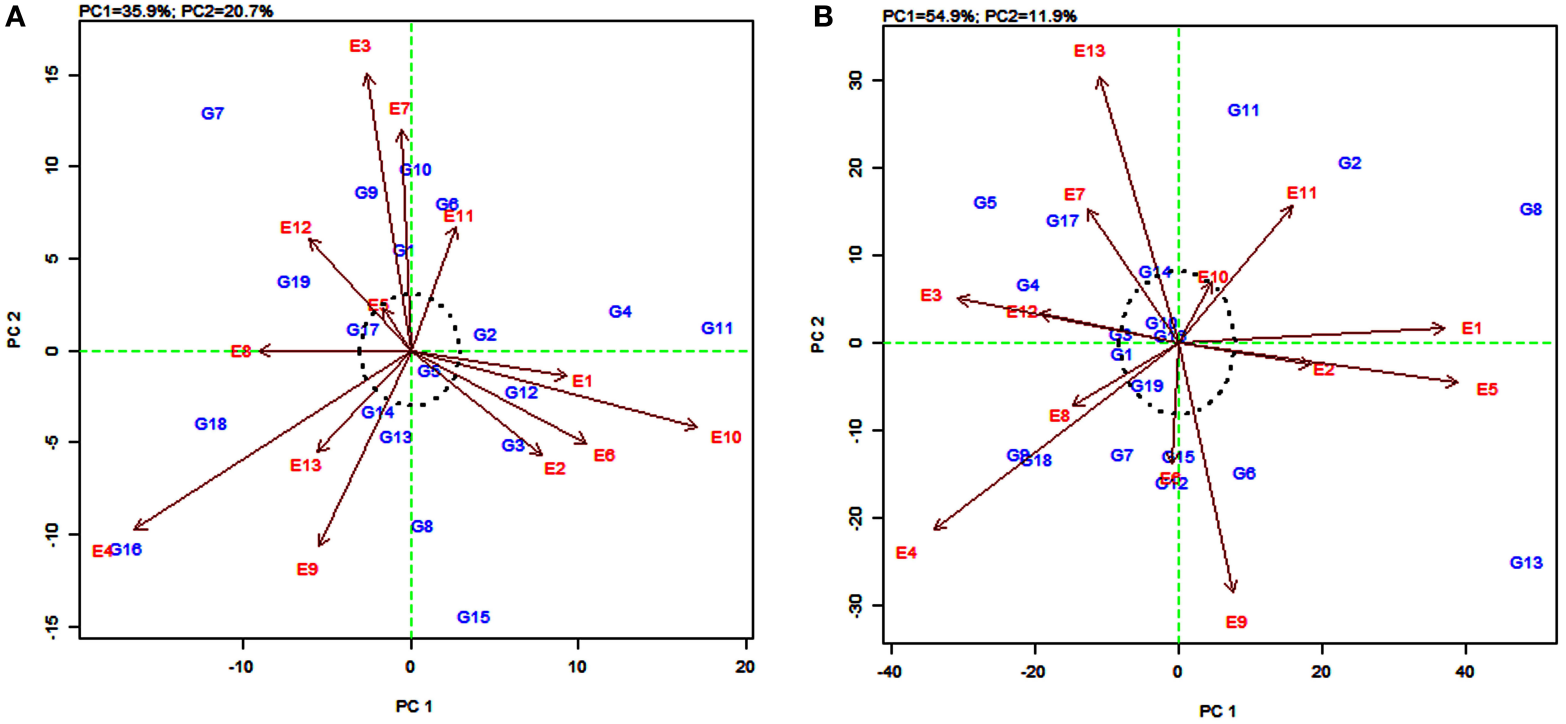
AMMI2 biplot showing two main axes of interaction (IPCA2 vs. IPCA1) for grain yield **(A)** and fodder yield **(B)** of 19 sorghum varieties tested across 13 environments.

In this study, for grain yield, the environments E4, E3, E10, and E9 were farthest from the origin and were the most discriminating but non-representative (unstable) while E5, E11, E2, E6, and E13 lied closest to the origin and contributed least to the G × E interaction (Figure 3A). They were the most representative (stable) environments but with poor discriminating ability. Sorghum varieties PYPS 11, PYPS 15, PYPS 10, and PYPS 7 were more responsive since they were away from the origin, whereas PYPS 5, PYPS 2, PYPS 17, and PYPS 14 were closer to the origin, and hence they were less sensitive to environmental changes for grain yield (Figure 3A). For fodder yield, the environments E13, E9, E4, E5, and E1 were the most discriminating but non-representative (unstable) and E10, E8, and E6 were the most stable environments (Figure 3B). Sorghum varieties PYPS 13, PYPS 11, PYPS 8, and PYPS 2 were more responsive and the varieties PYPS 19, PYPS 16, PYPS 1, PYPS 14, and PYPS 3 were less sensitive to changes in the environment (Figure 3B).

### GGE Biplot Analysis

The GGE biplot developed by Yan et al. (2000) displays the genotype main effect (G) plus G × E interaction, which are the two sources of variation that are relevant to a cultivar evaluation. The which-won-where pattern first described by Yan et al. (2000) identifies the best performer for a site(s) and defines mega-environments (subregions) by selecting the superior genotypes for each mega-environment, thus effectively exploiting both genotypes and G × E interaction.

**(a) The which-won-where pattern**

The polygon view of the GGE biplot displays the “which-won-where” pattern by connecting the markers of the genotypes that are further away from the biplot origin such that all the other genotypes are contained in the polygon (Yan et al., 2000). Genotypes having the specific adaptive ability for a specific environment or group of environments were identified by using this pattern. The biplot is further divided into sectors delimited by the lines perpendicular to each side of the polygon. The genotypes in a sector are similar in performance compared to the genotypes in other sectors.

**Figure 4:**
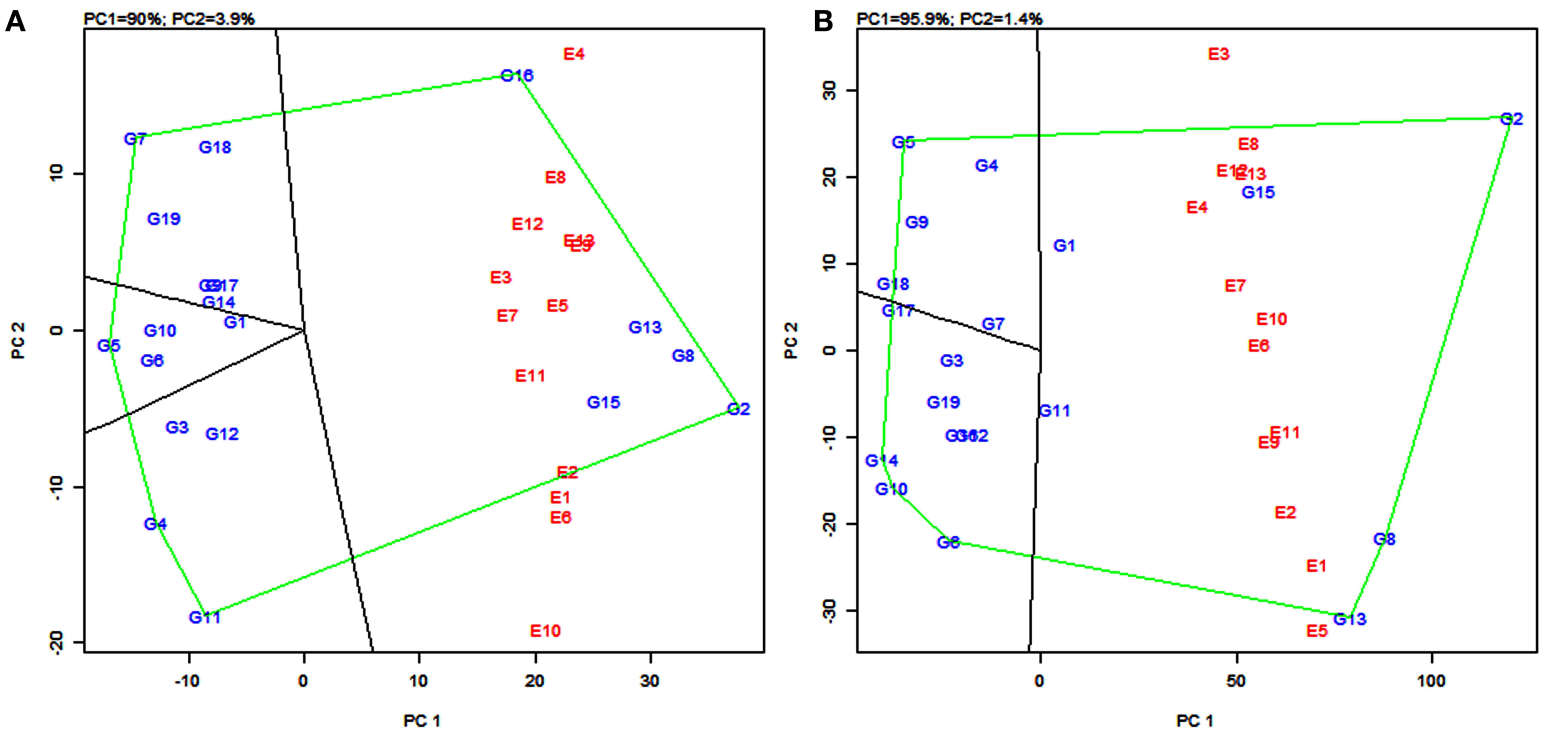
Polygon views of the genotype × environment interaction- (GGE-) biplot based on symmetrical scaling depicting “which-won-where” and mega-environment delineation for grain yield **(A)** and fodder yield **(B)** of 19 sorghum varieties tested across 13 environments.

In the present study, the biplot is divided into five sectors for grain yield (Figure 4A) and four sectors for fodder yield (Figure 4B). The varieties PYPS 2, PYPS 4, PYPS 5, PYPS 7, PYPS 11, and PYPS 16 were situated at the apex of the polygon, representing the highest grain yield and indicated superior genotypes (Figure 4A). Sorghum varieties PYPS 1, PYPS 9, PYPS 14, and PYPS 17 were closest to the center of origin indicated a low variation in the G × E interaction for grain yield (Figure 4A). The variety PYPS 16 was suitable for the three environments E4, E8, and E12. The varieties PYPS 2, PYPS 8, PYPS 13, and PYPS 15 were suitable for the remaining 10 environments E1, E2, E3, E5, E6, E7, E9, E10, E11, and E13 for grain yield (Figure 4A). The varieties PYPS 2, PYPS 5, PYPS 6, PYPS 10, PYPS 13, and PYPS 14 were situated at the apex of the polygon, representing the highest fodder yield and indicated superior genotypes (Figure 4B). Sorghum variety PYPS 2, PYPS 15, and PYPS 1 were suitable for E2, E3, E4, E6, E7, E8, E10, E12, and E13 for fodder yield. The varieties PYPS 8, PYPS 13, and PYPS 11 were suitable for environments E1, E2, E5, E9, and E11 (Figure 4B). The varieties PYPS 1, PYPS 3, PYPS 7, and PYPS 11 were closest to the center of the origin indicated a low variation in the G × E interaction for fodder yield (Figure 4B).

**(b) Mega-environments**

In the GGE biplot for grain yield, the five lines (rays) divided the biplot into five sectors. Environments were present in four sectors and these were considered as four mega-environments (Figure 4A) and the superior genotypes for each mega-environment were positioned at the vertex. The GGE biplot for grain yield resulted in four mega-environments *viz.*, first, a mega-environment comprising E8 and E12 with PYPS 16 as the best performing variety; second, a mega-environment comprising E9, E13, E3, E7, E5, E11, and E2 with PYPS 2 performing the best; third, a mega-environment comprising of E1, E6, and E10, where once again, PYPS 2 was the best performing variety; and fourth, a mega-environment with only one environment E4. Sorghum varieties PYPS 4, PYPS 5, PYPS 7, and PYPS 11 were located at the vertices in the sectors that did not show any environment, indicating that these genotypes were not superior in any of the mega-environments (Figure 4A).

Similarly, in the GGE biplot for fodder yield, four mega-environments were identified *viz.*, the first mega-environment comprising E4, E6, E7, E8, E10, E12, and E13 with PYPS 2 as the best performing variety; the second mega-environment comprising E1, E2, E9, and E11 with PYPS 8 and PYPS 13 as the best performing varieties (Figure 4B). The third and fourth mega-environment consisted of the single environment of E3 and E5, respectively, suitable for PYPS 2 and PYPS 13. Sorghum varieties PYPS 5, PYPS 6, PYPS 10, and PYPS 14 were located at the vertices in the sectors that did not show any environment, and hence they were not suitable for any mega-environment (Figure 4B).

In addition, the GGE biplot was used to graphically estimate the pattern of environments and discriminate the genotypes (Yan et al., 2000) based on the environment-focused scaling and genotype-focused scaling.

### GGE Biplot of Environment View for Yield

**Figure 5:**
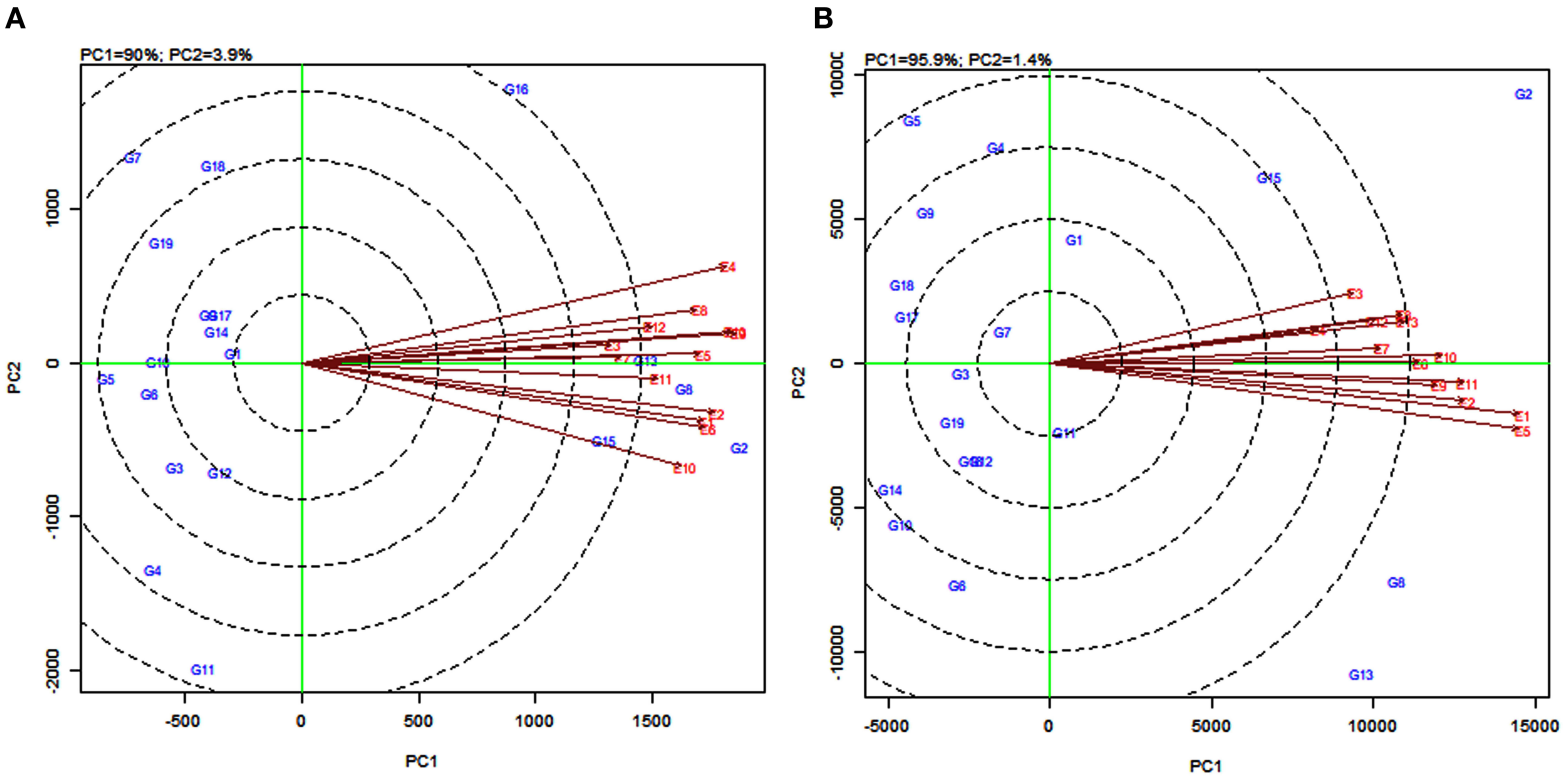
GGE biplot of the correlation among 13 tested environments for grain yield **(A)** and fodder yield **(B)** of 19 sorghum varieties.

The environmentally centered GGE biplot was used to estimate the pattern of environments for grain yield (Figure 5A) and fodder yield (Figure 5B). To compare the relationship between the environments, lines were drawn to connect the test environments to the biplot origin as environment vectors. The angle cosine between the two environments was used to determine the correlation between them (Dehghani et al., 2010). For grain yield, the angles between the vectors of the majority of the environments were acute, with few overlapping with one another, indicating a positive correlation (Figure 5A). For example, there was an overlapping between the vectors for the environments E3, E9, and E13, and also between E5 and E7. The presence of a wide angle between E4 and E10 indicated that they were negatively correlated and were not similar (Figure 5A). Similarly, for fodder yield, the presence of small angles between the vectors for an environment indicated that the tested environments were similar (Figure 5B). The widest angle between the vector of E3 and E5 suggested a dissimilarity between these two environments.

### GGE Biplot of Genotype View for Grain Yield

**Figure 6:**
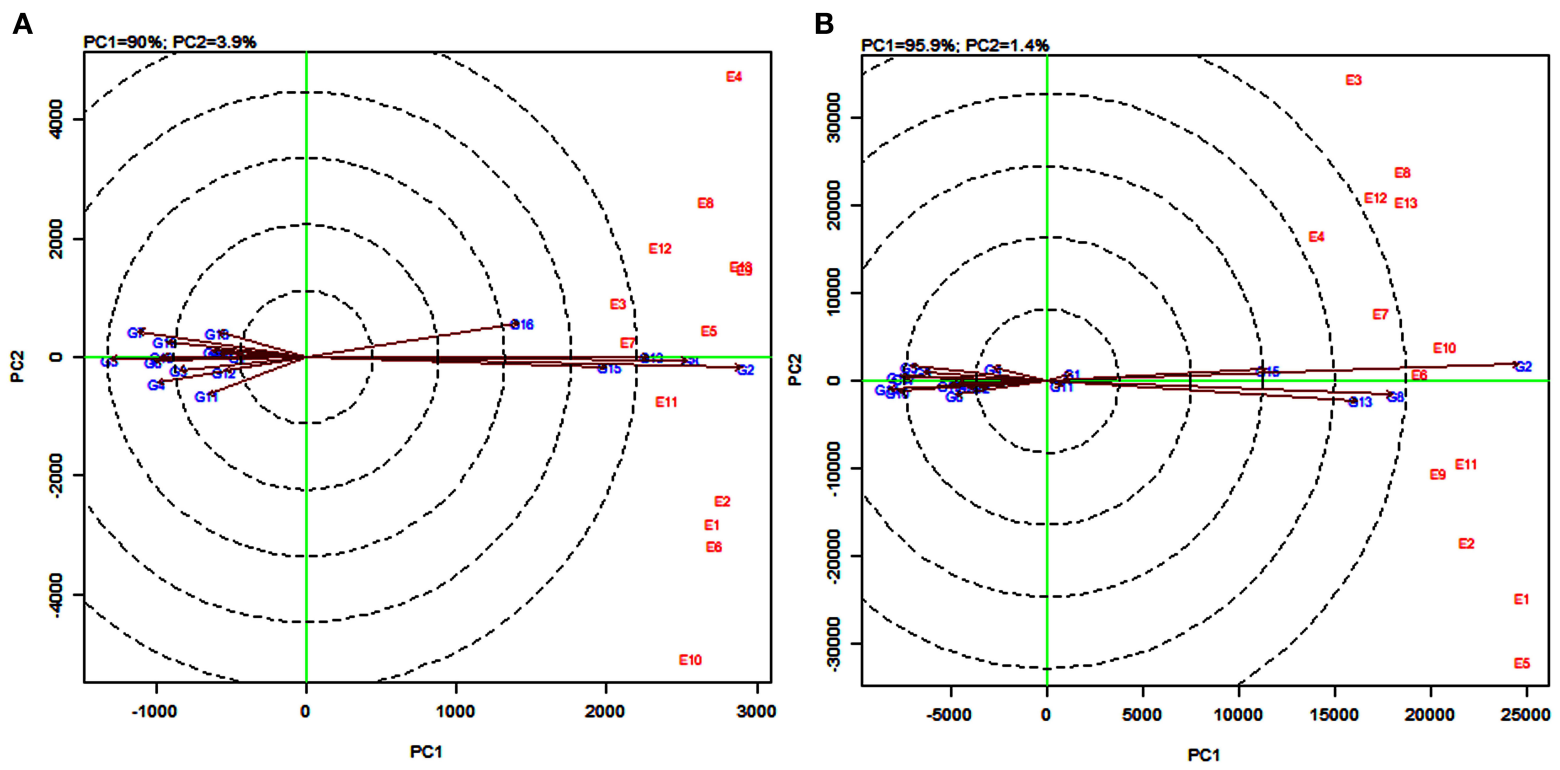
GGE biplot of genotype-focused scaling for discriminating 19 sorghum varieties tested across 13 environments for grain yield **(A)** and fodder yield **(B)**.

The vector view of GGE biplot in the genotype-focused scaling measured their dissimilarity in discriminating the genotypes (Kumar et al., 2021). For grain yield, sorghum varieties PYPS1, PYPS 6, PYPS 7, PYPS 9, PYPS 10, PYPS 17, PYPS 18, and PYPS 19 showed the same group position. The varieties PYPS 3, PYPS 4, PYPS 5, PYPS 8, PYPS 11, and PYPS 12 fell in the same group. Likewise, the varieties PYPS 2, PYPS 13, PYPS 14, and PYPS 15 were in the same group. One sorghum variety PYPS 16 with a distinct group was discriminating suggesting dissimilarity with other groups (Figure 6A). For fodder yield, PYPS 1, PYPS 2, and PYPS 15 showed the same group position (Figure 6B). The varieties PYPS 8, PYPS 11, and PYPS 13 showed the same group position with the remaining varieties in two different group positions.

### GGE Biplot on Environment for Comparing Environments With an Ideal Environment

**Figure 7:**
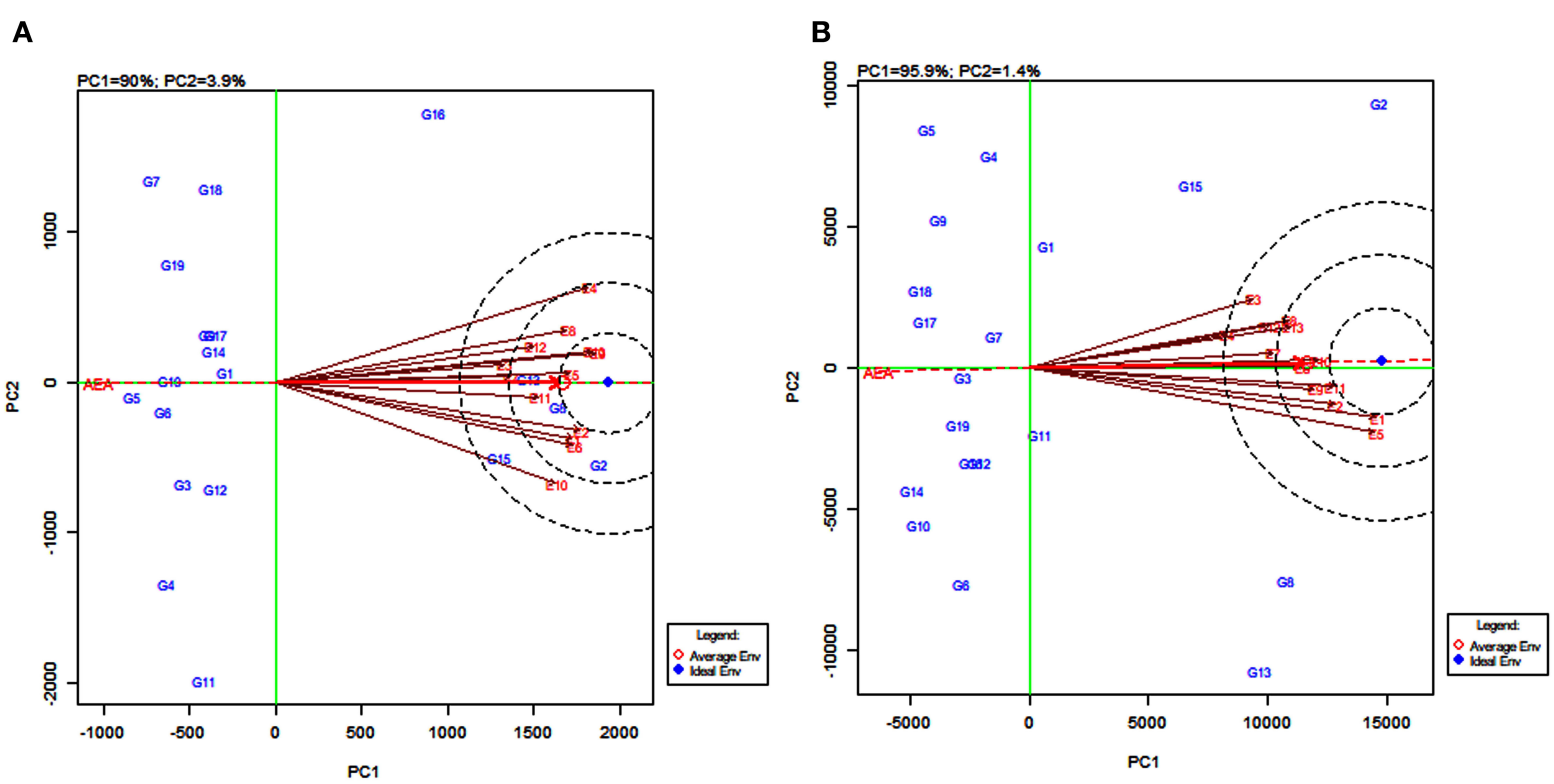
GGE biplot of environment-focused scaling for comparing 13 test environments with an ideal environment for 19 sorghum varieties for grain yield **(A)** and fodder yield **(B)**.

Discriminating ability and representativeness of the testing environments are important measures of the GGE biplot. The concentric circles in Figure 7 help us in visualizing the length of the environment vectors, which are a measure of the discriminating ability of the environments and the SD within the respective environments (Yan and Tinker, 2006). The average environment that is drawn as a small circle at the end of the arrow (Figures 7A,B) has the average coordinates of all test environments, and the average environment axis (AEA) is the line passing through the average environment and the biplot origin. A test environment showing a smaller angle with the AEA is more representative than the other test environments (Yan and Rajcan, 2002).

For grain yield, the environments E4, E9, and E13 were the most discriminating genotypes, very closely followed by E1, E2, E6, and E10. The environments E3 and E7, with the shortest vectors from the origin, provided little or no information about the genotype difference and were considered as non-discriminative environments (Figure 7A). Based on the angle of the environment with AEA, the environments E5 and E11 were the most representative whereas E4 and E10 were the least representatives for grain yield. For fodder yield, the environments E1 and E5 were the most discriminating genotype while E2, E9, E10, and E11 were moderately discriminating. The environment E4 followed by E3 and E7 were considered as non-discriminative environments. Further, the environments E10 and E6 were the most representative whereas E3 and E5 were the least representatives for fodder yield (Figure 7B). The environments E5, E9, and E13 (Figure 7A) and the environments E11 and E12 (Figure 7B) located in the first concentric circles were identified as the most ideal environments for obtaining high grain and fodder yields, respectively. The evaluation in these environments maximized the observed genotypic variation among the 19 tested sorghum varieties.

### GGE Biplot of Stability and Mean Performance of Genotypes Across Average Environments

**Figure 8:**
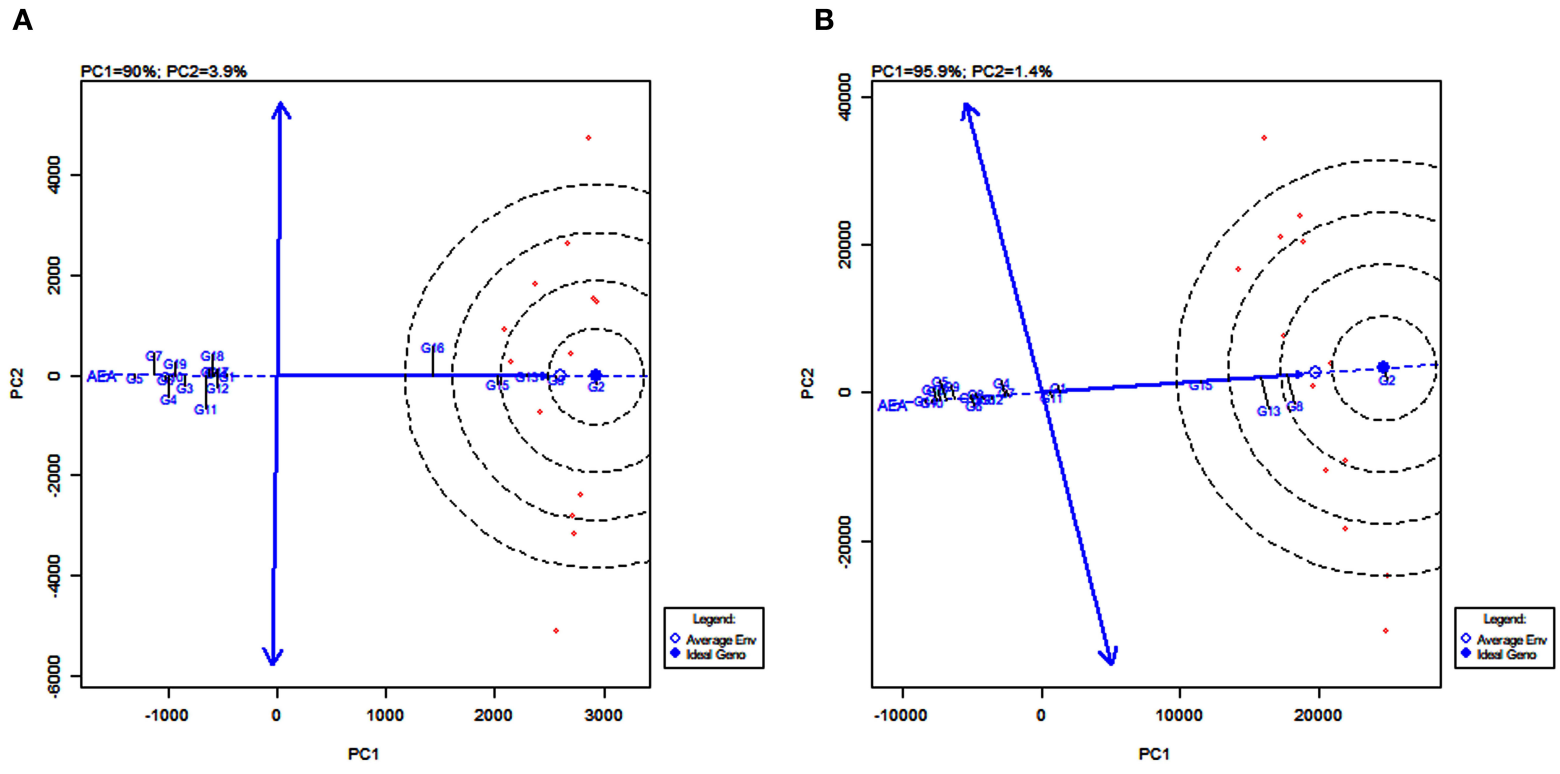
GGE biplot of stability and mean performance of 19 sorghum varieties across average environments of 13 test environments for grain yield **(A)** and fodder yield **(B)**.

The line passing through the biplot origin and the average environment with a single arrow is the AEA. Projections of genotype markers to the AEA depict the mean yield of genotypes (Figure 8). For grain yield, PYPS 2 was the high-yielding variety and PYPS 5 was the lowest yielding variety (Figure 8A). For fodder yield, PYPS 2 was once again the high-yielding variety and PYPS 10 was the lowest yielding variety (Figure 8B). A double arrowed line passing through the biplot origin and perpendicular to the AEA abscissa is the AEA ordinate (Figure 8). A greater projection onto AEA ordinate regardless of the direction means a greater stability. Accordingly, sorghum varieties PYPS 2, PYPS 5, PYPS 8, PYPS 13, PYPS 14, and PYPS 17 with shorter projections over the environments were stable, and the varieties PYPS 11 and PYPS 16 were unstable for grain yield (Figure 8A). For fodder yield, PYPS 2, PYPS 10, PYPS 15, and PYPS 16 were mostly stable over the environments, and the varieties PYPS 5, PYPS 8, and PYPS 13 were unstable (Figure 8B).

#### AMMI and GGE Analyses for Grain Mold Tolerance

Sorghum varieties PYPS 8 and PYPS 13 recorded low disease scores of 3.71 and 3.97, respectively, for grain mold incidence. Further, these varieties showed low IPCA1 values (0.01 and 0, respectively) and ASVs (0.04 and 0.09, respectively), and hence they were considered as the most stable varieties against grain mold disease (Table 7, Supplementary Table 2). Sorghum variety PYPS 2 showed less incidence of grain mold with a score of 3.92. However, the ASV was relatively high at 0.60, and hence it was moderately stable.

**Table 7:** Disease reaction scores against grain mold, IPCA scores, and ASV values of 19 sorghum genotypes over three environments from 2016 to 2018.

**S. No**.	**Genotype/Environment**	**Score**	**PC1**	**PC2**	**ASV**
1	PYPS-1	4.52	−0.07	0.08	0.01
2	PYPS-2	3.92	0.24	0.06	0.60
3	PYPS-3	4.50	0.02	0.11	0.11
4	PYPS-4	4.10	−0.16	−0.11	0.40
5	PYPS-5	4.51	0.03	0.07	0.09
6	PYPS-6	4.73	−0.13	0.03	0.32
7	PYPS-7	4.18	−0.07	0.06	0.18
8	PYPS-8	3.71	0.01	−0.04	0.04
9	PYPS-9	4.78	0.00	−0.09	0.09
10	PYPS-10	5.10	−0.13	0.14	0.37
11	PYPS-11	3.65	0.09	0.07	0.23
12	PYPS-12	4.80	−0.07	0.11	0.06
13	PYPS-13	3.97	0.00	−0.09	0.09
14	PYPS-14	5.32	0.03	0.02	0.07
15	PYPS-15	4.54	−0.03	−0.06	0.08
16	PYPS-16	5.00	0.01	−0.02	0.02
17	PYPS-17	4.74	0.20	−0.03	0.50
18	PYPS-18	4.73	−0.12	−0.07	0.72
19	PYPS-19	4.39	0.09	−0.04	0.32
20	SPV 462 (Susceptible check)	8.33	0.02	−0.13	0.24
21	IS 8545 (Resistant check)	4.04	0.03	−0.03	0.06
	E1	4.66	0.30	−0.17	0.33
	E2	4.63	0.04	0.29	0.29
	E3	4.64	−0.34	−0.12	0.85
	LSD (0.05)	2.54			

*IPCA, Interaction principal component axis; ASV, AMMI stability value; LSD, Least significant difference*.

*Disease Score between 3.0 and 5.0 with 6–30% grain mold incidence is considered as tolerant, disease score 6.0–9.0 with 31–100% grain mold incidence and identified as susceptible*.

The AMMI biplot revealed that the varieties PYPS 2, PYPS 11, and PYPS 19 had a low grain mold incidence and were moderately stable (Figure 5A). The varieties PYPS 4 and PYPS 7 had low grain mold disease but were unstable. The variety PYPS 14 and the susceptible check SPV 462 were stable with a high grain mold incidence. The GGE biplot graphic analyses of 19 sorghum varieties tested at three environments revealed the magnitude of the interaction of each genotype and environment for grain mold incidence (Figure 5B). PYPS 2, PYPS 4, PYPS 8, and PYPS 11 were the most stable varieties for E2 with a low grain mold incidence followed by the varieties PYPS 7 and PYPS 13. The susceptible check SPV 462, though suitable for environment E2, recorded a high incidence of grain mold.

### Nutrient Composition of Sorghum Varieties

The starch, sugar, protein, Fe, and Zn contents varied among the 19 tested sorghum varieties (Table 8). The starch content ranges from 32.11% (PYPS 16) to 57.09% (PYPS 13). The total sugar content among the tested varieties varied from 5.25% (PYPS 9) to 14.93% (PYPS 15). The highest percentage of total protein content was encountered in the grains of PYPS 2 (14.52%) and PYPS 8 (14.13%) whereas the lowest one was demonstrated in PYPS 15 (8.78%). There were significant (*p* < 0.05) differences in the total Fe content among the tested sorghum varieties, with the highest total Fe content in PYPS 4 (12.75 mg) followed by PYPS 2 (10.75 mg) and the lowest in PYPS 15 (4.31 mg) and PYPS 18 (4.40 mg). Significant differences were also found among the sorghum varieties for the total Zn content. The varieties PYPS 1 (3.40 mg), PYPS 8 (3.40 mg), and PYPS 9 (3.30 mg) had the highest Zn content while the varieties PYPS 15 (1.80 mg), PYPS 5 (1.90 mg), PYPS 7 (1.90 mg) recorded the lowest Zn content.

**Table 8:** Nutrient composition of sorghum genotypes collected from E1 (Palem, Telangana, India) in 2018.

**S. No**.	**Genotype**	**Starch**	**Sugar**	**Fe (mg/100 g)**	**Zn (mg/100 g)**	**Protein (%)**
1	PYPS-1	49.73	8.76	5.60	3.40	12.32
2	PYPS-2	53.93	10.01	10.70	2.80	14.52
3	PYPS-3	38.44	12.68	5.50	2.40	9.78
4	PYPS-4	48.61	14.09	12.70	2.90	10.30
5	PYPS-5	53.88	11.43	5.40	1.90	10.52
6	PYPS-6	43.50	9.13	6.20	2.20	9.63
7	PYPS-7	45.13	12.18	7.40	1.90	9.25
8	PYPS-8	48.77	9.67	6.60	3.40	13.26
9	PYPS-9	52.86	5.25	6.00	3.30	14.13
10	PYPS-10	39.46	12.30	7.80	2.50	9.58
11	PYPS-11	45.43	9.84	5.80	2.70	10.73
12	PYPS-12	46.50	9.95	5.30	2.10	10.95
13	PYPS-13	57.09	8.82	4.90	2.40	10.66
14	PYPS-14	43.05	6.77	5.40	3.00	10.52
15	PYPS-15	40.16	14.93	4.40	1.80	8.78
16	PYPS-16	32.11	13.59	6.40	2.90	10.77
17	PYPS-17	56.38	6.98	5.40	2.00	11.28
18	PYPS-18	38.29	9.77	4.40	2.30	10.60
19	PYPS-19	45.20	9.60	6.70	2.80	10.72
	LSD	7.21	2.84	2.14	0.64	2.15
	CV%	14.71	11.41	8.75	7.86	14.24
	Range	24.98	9.68	8.30	1.6	5.74

*LSD, Least significant difference; CV, Coefficient of variation*.

## Discussion

In this study, a total of 108 landraces were collected, and 36 superior landraces of 108 landraces were identified. About 11 of these landraces were used to develop 19 sorghum varieties. Previous reports (Tesso et al., 2008; Adugna, 2014; Abraha et al., 2015; Amelework et al., 2016; Wondimu et al., 2020) have identified sorghum landraces with a wide range of variations that could provide new sources of tolerance and highly contrasting lines for use in future breeding programs. The landraces used in the present study were collected from the interior parts of central and southern India where they might have evolved in response to diverse agroecological zones and farming systems practiced in those regions and were better adapted to the low input and marginal cultivation conditions of these areas coupled with a frequent occurrence of grain mold disease. The 13 rainfed locations tested in this study represented the rainfed dryland conditions and were characterized by a complex climate that is largely semiarid and dry subhumid, with a short wet season followed by a long dry season. Sorghum cultivated in these regions is prone to highly erratic rainfall (spatially and temporally), with a strong risk of dry spells at critical growth stages and heavy rains at the grain maturity stage. The present study identified 36 superior performing landraces, cultivated in water-stress conditions and had a tolerance to grain mold due to an indirect selection for associated traits such as panicle shape, grain color, seed compactness, etc. over the years, which could be utilized to develop elite lines in sorghum breeding programs.

In the present study, the evaluation of 19 sorghum varieties derived from the superior landraces using AMMI and GGE biplots has demonstrated a higher contribution by the G × E interaction to the total variation than the genotypes suggesting that the environment had a high impact on the performance of the sorghum varieties for both grain yield and fodder yield. Even though the proportion of the environment is the largest for both grain and fodder yields, the genotypes and G × E interaction have a paramount importance for the genotype evaluation (Yan and Kang, 2003). These findings are in agreement with Reddy et al. (2014) and Al-Naggar et al. (2018) who reported the predominance of the environment's main effect as the source of variation in the multi-environment trials in sweet sorghum and grain sorghum, respectively. Abiotic factors such as soil moisture, pH, mineral availability along with the weather and biotic factors including natural pest and disease occurrence might have contributed to large variations in the yield performance of the genotypes. The potential of the genotypes could be more exploited if the best performing genotypes were identified for the specific environments.

The total variations (56.6, 66.8%) contributed by IPCA1 and IPCA2 explained the differential performance of genotypes for grain yield and fodder yield across the 13 environments. Because of their maximum contribution, IPCA1 and IPCA2 were used to plot a two-dimensional GGE biplot. Gauch (2013) suggested that the most accurate model for AMMI can be predicted by using the first two IPCAs. Several researchers used the first two IPCAs for the GGE biplot analysis because they explained a greater percentage of the G × E interaction for sorghum (Al-Naggar et al., 2018), barley (Vaezi et al., 2017), pigeonpea (Rao et al., 2020; Kumar et al., 2021), wheat (Verma et al., 2015), and maize (Solomon et al., 2008).

AMMI stability value, which is the quantitative stability value developed through the AMMI model by Purchase et al. (2000), has been considered as the most appropriate single method of describing the stability of genotypes (Naroui et al., 2020). Several studies have identified the genotypes with smaller ASV and better stability and those with high ASV but higher yields for specific adaptability in crops including bread wheat (Farshadfar, 2008), grain sorghum (Adugna, 2014), and finger millet (Lule et al., 2014). In the present study, the high yields and stability (low ASV) of varieties such as PYPS 2, PYPS 5, PYPS 8, and PYPS 13 might be attributed to the 11 parental landraces, which could offer potential new sources of genes for higher grain and fodder yields and stability. These genotypes merit further genetic studies for adaptation and physiological traits for dissecting the traits contributing to stability.

In this study, several stable sorghum varieties were identified for grain yield and fodder yield. For example, PYPS 8 was stable and high in yield for grain yield. However, most of the stable genotypes need not necessarily have the best yield performance (Mohammadi and Amri, 2007). Hence, GSI was used as a single criterion to classify stable genotypes. To our knowledge, this is the first study incorporating grain and fodder yields' mean, protein content, grain mold reaction, and stability index to identify high-yielding, high protein, grain mold tolerant, and stable sorghum varieties such as PYPS 1, PYPS 2, PYPS 8, PYPS 13, etc. In wheat, Singh et al. (2019) have used GSI to identify stable high-yielding genotypes in India.

Genotypic stability is crucial to grain yield and the best genotype needs to combine good yield and stable performance across a range of production environments. Based on the AMMI1 biplot, the current study identified the sorghum varieties with wide and specific adaptability for both grain yield and fodder yield. For example, sorghum variety PYPS 8 with a low absolute IPCA1 score and the above-average grain yield was stable, showing less-variable yield across the environments, thus making it a promising multilocation testing and validation. On the contrary, the varieties PYPS 1 and PYPS 10 performed consistently across locations but with below-average grain yields. The variety PYPS 2 showed specific adaptability to E1 and E10 for grain yield. Similarly, Al-Naggar et al. (2018) identified two grain sorghum B-lines with site-specific adaptability in Egypt.

**Figure 9:**
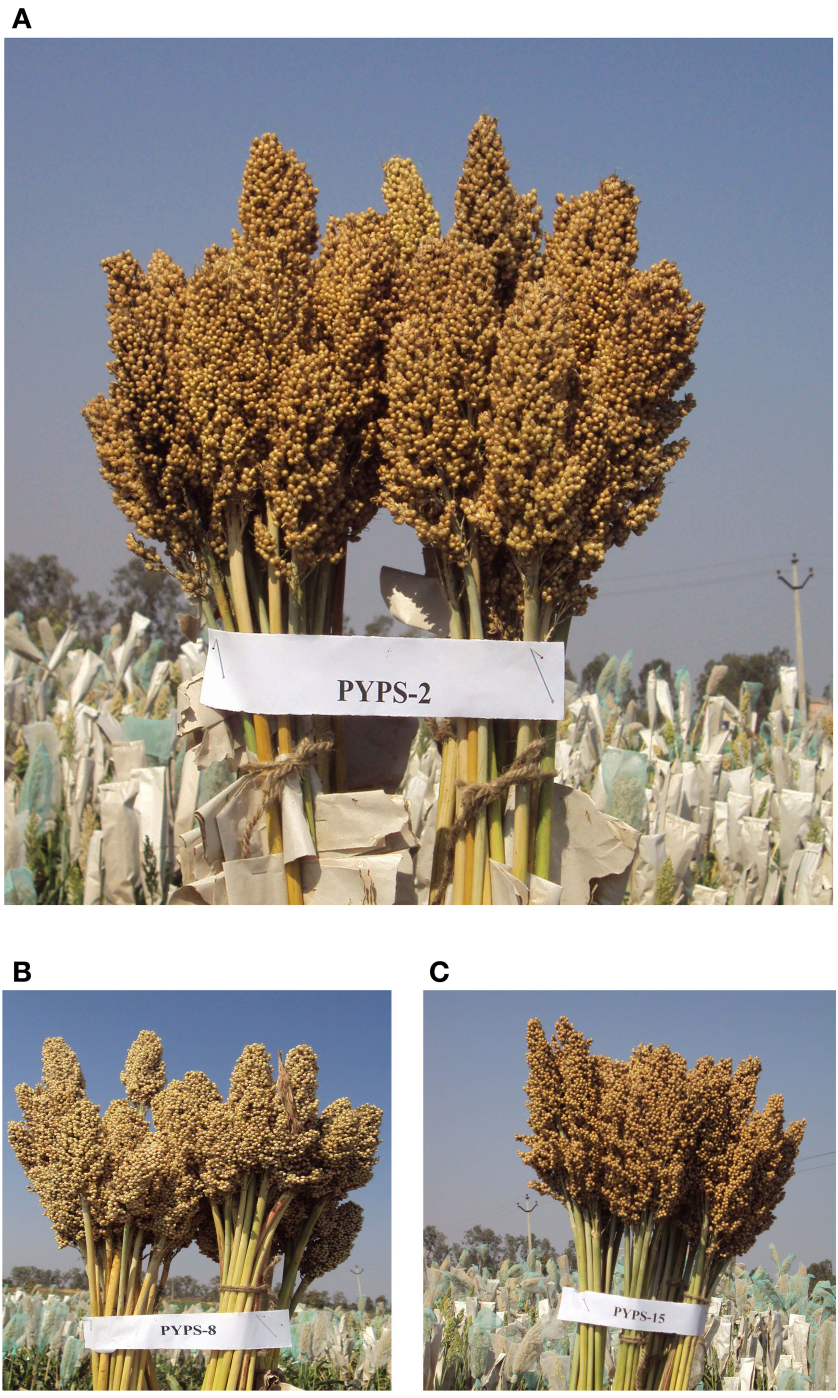
High-yielding, grain mold-tolerant, and protein-rich sorghum varieties developed from the superior landraces. **(A)** PYPS 2 (grain yield: 3,698 kg/ha and protein: 14.51%), **(B)** PYPS 8 (grain yield: 3,584 kg/ha and protein: 14.13%), and **(C)** PYPS 15 (grain yield: 3,432 kg/ha and protein: 8.78%).

Interestingly, the varieties differed in their grain yield and fodder yield performances across locations. For example, the variety PYPS 15 showed specific adaptation to environments E1 and E10 for high grain yield (3,432 kg/ha) but was the most widely adaptable variety for fodder yield (16,955 kg/ha). Sorghum varieties PYPS 2, PYPS 8, PYPS 13, and PYPS 15 were considered as the best dual purpose cultivars (Figure 9) due to their higher grain and fodder yields (3,698, 2,0586 kg/ha; 3,584, 18,632 kg/ha; 3,514, 18,122 kg/ha; 3,432, 16,955 kg/ha) whereas PYPS 16 and PYPS 11 were the best varieties exclusively for grain (3,268 kg/ha) and fodder (13,895 kg/ha).

The relationship between the testing environments was graphically evaluated by using the angles between the vectors. The presence of wide obtuse angles between E5 and E12 with E2 suggested a negative correlation or strong crossover G × E interaction for grain yield (Yan and Tinker, 2006). This indicated that the genotypes performing better in one environment would perform poorly in another environment. Closer relationships among the locations depicted by small cosine angles (<90°) indicated the non-existence of a crossover G × E interaction suggesting that the ranking of genotypes does not change from environment to environment. The environments E5 and E12 and the environments E3 and E7 fall under the latter. The present study indicated a mixture of crossover and non-crossover types of G × E interaction, which has been reported in various studies (Rakshit et al., 2012; Naroui et al., 2013; Aruna et al., 2015). Furthermore, eliminating similar environments from multilocation trials of sorghum will help in the optimal utilization of resources.

In the polygon view of the GGE biplot derived from the first two main PC, PC1 refers to the yield ratio, associated with genotypic characteristics and PC2 refers to the yield related to the G × E interaction (Yan et al., 2007; Yan and Holland, 2010). In the present study, the contribution of the first two PCs toward 99.9 and 95.9% of the variability for grain yield and fodder yield, respectively, justified the use of the GGE biplot to effectively interpret the variability in the multi-environment data. The 13 tested environments in this study contributed to 35.5% of the total variation in grain yield and 28.9% in fodder yield. Mushayi et al. (2020) reported as much as 63% of the variation being explained by location in grain yield for maize. In this study, the highest grain yielding variety PYPS 2 performed best at E2, E1, E11, E6, and E10. These environments are in complete contrast to the best yielding environments E3, E8, E12, and E13 for high fodder yield. Several authors identified high-yielding genotypes for grain sorghum (Rakshit et al., 2012; Al-Naggar et al., 2018), forage sorghum (Aruna et al., 2015), and sweet sorghum (Rono et al., 2016).

In the GGE biplot analysis, partitioning of the test locations into meaningful mega-environments is the best approach to exploit the positive G × E interaction (Yan and Tinker, 2006). In the present study, the test locations were divided into a total of four mega-environments for grain yield and four for fodder yield. The varieties performed differently across the mega-environments for grain yield and fodder yield. For example, the variety PYPS 2 was the best for the two mega-environments for high grain yield and fodder yield and PYPS 5 was not suitable for any mega-environment suggesting that different genotypes should be deployed for each mega-environment to achieve optimal adaptation.

Based on the AMMI analysis, PYPS 8 and PYPS 13 were identified as the most stable sorghum varieties with a low grain mold incidence. The AMMI and GGE biplot methods were used to identify resistance sources to different diseases in multiple locations and also high-yielding stable genotypes in wheat, groundnut, mungbean, melon, etc. (Akcura et al., 2017; Chaudhari et al., 2019; Naroui et al., 2020; Tollo et al., 2020). In a previous study, Diatta et al. (2019) evaluated five sorghum hybrids along with their parental lines and reported a significant G × E interaction for grain yield. However, the G × E interaction was not significant for panicle mold infestation. Grain mold is a complex disease whose incidence is governed by a host resistance and an environment. Grain mold resistance was correlated with open panicles, long glumes, a greater glume coverage length, and area (Sharma et al., 2010). Loose panicle sorghum is more likely to be resistant to grain mold because the compact heads hold more moisture that favors disease development. Weather factors, particularly relative humidity play a dominant and determining role in grain mold severity. The wet weather condition following flowering is necessary for the grain mold development. The longer the duration of wetness on grain surface, the greater is the incidence of grain mold (Das et al., 2020). Photoperiod-sensitive cultivars that mature after the rains often escape grain mold infection (Patted et al., 2011). Sorghum cultivars with a white grain are more vulnerable to grain mold than those with a brown and red grain pericarp. In the present study, the varieties PYPS 8 and PYPS 13, which showed a low grain mold incidence, had a brown and yellow pericarp, respectively. Their parental lines *viz.*, PSLRC 8, PSLCRC 9, PSLRC 2, and PSLRC 21 were characterized by a brown and yellow pericarp with high tannin levels, which might have contributed to grain mold tolerance. Furthermore, their panicles were semi-compact to loose a trait important for grain mold tolerance due to non-retention of moisture and better air circulation (Glueck et al., 1977; Rao et al., 1999; Patted et al., 2011). Glume length and area of coverage over the grain is related to the grain mold escape as the grains are protected from the exposure to rain. Patted et al. (2011) reported that sorghum progenies with a very long-to-long glume coverage escaped grain mold tolerance. They also reported that black- and red-colored glumes were moderately resistant to grain mold due to the presence of tannins, which inhibit the growth of saprophytic fungi, and thus reducing a grain mold incidence.

An analysis of nutrient composition revealed a mean starch content of 46.04% that is lower than the average content varying from 56 to 73% (Ratnavathi and Patil, 2013). The protein content ranging from 10.4 to 10.62% was reported in sorghum germplasm collections (Weckwerth et al., 2020). Other studies in sorghum have reported the protein content of 9–11% (Elbashir et al., 2008; Ahmed et al., 2014). Abdelhalim et al. (2021) reported the protein content ranging from 6.3 to 12.5% among the five landraces evaluated for their potential in biofortification. The protein content of the three varieties *viz.*, PYPS 2 (14.52%), PYPS 9 (14.13), and PYPS 8 (13.26%) tested in this study is higher than that of sorghum landraces reported by Abdelhalim et al. (2021). The high protein content of the genotypes in this study suggested the possibility of a similar feature in the parent landraces PSLRC 2, PSLRC 3, PSLRC 4, PSLRC 8, PSLRC 9, and PSLRC 10. These landraces, in addition to the genotypes PYPS 2, PYPS 8, and PYPS 9, offer a significant source of new genetic materials, as well as fortified ingredients for enhancing the nutritional value of sorghum grains. One nutritional constraint to the use of sorghum as food is the poor digestibility of sorghum proteins in cooking (Tesso et al., 2008). Duressa et al. (2020) have reported sorghum accessions with a protein content as high as 15% but with different digestibility percentages (a measure of the susceptibility of protein to proteolysis). For example, the sorghum accession SC663 with a protein content of 15.5% had a high protein digestibility of 70.77%. On the contrary, the sorghum accession SC25 with a protein content of 14.8% had a protein digestibility of 49.73%. Hence, it is important that the sorghum varieties with a high protein content are evaluated for protein digestibility to identify the genotypes with high protein availability.

In the present study, the varieties varied in their grain Fe and Zn concentrations. Phuke et al. (2017) reported a highly significant G × E interaction for Fe and Zn levels in sorghum recombinant inbred lines. They found a significant variation in Fe and Zn across the environments and a significant positive correlation between the Fe and Zn concentrations. While the varied concentrations of Fe and Zn in the present study might be genetically controlled, the effect of the environment and the possible G × E interaction need to be investigated by multi-environment testing of the sorghum genotypes. Shegro et al. (2012) also reported a diversity in nutritional composition among the sorghum landraces and identified the accessions with high concentrations of proteins and minerals (Fe, Fe, Ca, K, Mn, P, and Mg). The variations in mineral contents of the 19 sorghum genotypes in this study may be due to genotype, mineral concentrations in the soil as well as translocation rates of the elements from the soil, as well as environmental factors such as temperature and rainfall or an inherent ability of the genotypes to absorb the nutrients from the soil (Shegro et al., 2012). The assessment of a phenotypic correlation among the protein, starch, sugar, and mineral compositions of the genotypes in this study might have implications for the sorghum crop improvement in relation to human nutrition and livestock feed. Weckwerth et al. (2020) have suggested the use of genomic selection (GS) using genome-wide DNA markers in crop breeding programs to target multiple and complex traits. This can be deployed in a sorghum breeding program to simultaneously target yield, drought tolerance, and nutritional composition including protein, starch, Fe, and Zn. Not just for sorghum, but such an approach integrating GS with an environment-dependent PANOMICS analysis can improve the productivity, biotic and abiotic stress tolerance, and nutritional value of several crops including pigeonpea, groundnut, chickpea, etc. (Weckwerth et al., 2020).

In cereals, the nutritional quality and end use properties are linked to arabinoxylans (AX) that are the major polymers present in the cell walls of grains (Saulnier et al., 2007). Though wheat AX has been extensively reviewed (Saulnier et al., 2007), limited information is available on sorghum AX concerning the amount, composition, and expression. Nandini and Salimath (2001) reported that the structural features of AX are linked with a good flatbread-making quality in sorghum. PYPS-2, the high yielding, protein-rich, and grain mold-tolerant genotype has a high consumer preference owing to good flatbread-making quality (Jaisimha, 2019), which might be linked to the structure and/or amount of AX. Though the expression is mainly genetically controlled, a positive relationship with the amount of AX and drought has been reported in wheat (Coles et al., 1997). As demonstrated in this study, the 36 landraces and the 19 genotypes were evaluated under rainfed situations where prolonged drought stress is a common occurrence, which may have influenced the AX expression *vis-à-vis* flat bread-making quality. The evaluation of sorghum genotypes for their AX amount and expression in different environments might help in identifying stable and adaptable cultivars with enhanced nutritional quality. To achieve this, research may first be reoriented toward the understanding of the AX biosynthesis, the identification of the candidate genes, and exploitation of the variation toward the development of new cultivars with improved nutritional quality and processing properties.

## Conclusion

The present study reports the development of an elite sorghum breeding material using locally adapted landraces through pedigree breeding for the grain and fodder yield. The study has identified stable environments for grain yield (E5) and fodder yield (E11), which suggest that testing of initial hybrids and varietal trials in these two environments is more discriminating and rewarding to save resources in Telangana, India. The study showed the potential of the collected landraces for the development of high-yielding sorghum varieties suitable for rainfed cultivation in India and spill-over to African subcontinents. The study demonstrated a scope for the utilization of these diverse, locally adapted landraces that have been evolved through a continuous selection by the farmers in varied-agro-climatic zones under low input conditions to develop climate-resilient sorghum cultivars and ultimately contribute to healthy, global food, and feed security.

## Data Availability Statement

The original contributions presented in the study are included in the article/Supplementary Material, further inquiries can be directed to the corresponding author/s.

## Author Contributions

MN, RV, MG, and MP designed the experiment. MN and RV carried out the experiments. MN, RV, MG, and SG analyzed the data. CS, SM, SG, and HK conducted the field experiments with logistical support by MV, KA, and RJ. MS helped in nutrient analysis. MN, RV, and MG wrote the draft manuscript. All authors made contributions toward the compilation of the draft and approved the final manuscript.

## Conflict of Interest

The authors declare that the research was conducted in the absence of any commercial or financial relationships that could be construed as a potential conflict of interest.
