# Supplementary material for: Harnessing Sorghum Landraces to Breed High-Yielding, Grain Mold-Tolerant Cultivars With High Protein for Drought-Prone Environments
Source: Front Plant Sci. 2021 Jun 30;12:659874. doi: 10.3389/fpls.2021.659874 (PMC8279770; doi:10.3389/fpls.2021.659874)
Supplement: Supplementary file 1 [file Data_Sheet_1.PDF]

SUPPLEMENTARY TABLE S1: Details of Sorghum landraces collected from various parts of southern and central India during 2008

S. No.	Local name	State	Location/ District	Accession Identifier given by ICAR-NBPGR	Days to 50% Flowering	Panicle compactness	Grain Color	Accession Identifier given under the project
1	Andhol jonna	Telangana	Medak	IS-5281	68	Semi compact	Yellow	
2	Chilaka jonna	Telangana	Mahabubnagar	IS-36079	62	Semi compact	Yellow	
3	Cheruku patcha jonna	Andhra Pradesh	Nandyal	IS-1132	60	Compact	Yellow	
4	Chinna sai jonna	Telangana	Medak	IS-5273	62	Compact	Yellow	
5	Chitta patcha jonna	Andhra Pradesh	Kurnool	IS-5065	66	Semi compact	Yellow	PSLRC 7
6	Garibi jowar	Telangana	Adilabad	IS-33661	60	Semi compact	Yellow	
7	Getu jonna	Telangana	Mahabubnagar	IS-36081	66	Compact	Yellow	
8	Gundu patch jonna	Andhra Pradesh	Nandyal	IS-1138	71	Semi compact	Yellow	PSLRC 1
9	Gundu patcha jonna	Andhra Pradesh	Kodumuru	IS-21979	68	Semi compact	Yellow	PSLRC 2
10	Jadia dholio	Telangana	Adilabad	IS-1149	58	Compact	Yellow	
11	Jonna saner	Andhra Pradesh	Anantapur	IS-5095	67	Semi compact	Yellow	
12	Jowari langpur	Telangana	Adilabad	IS-5244	62	Semi compact	Yellow	PSLRC 14
13	Karimdevpeth jonna	Telangana	Warangal	IS-5226	62	Compact	Yellow	

S. No.	Local name	State	Location/ District	Accession Identifier given by ICAR-NBPGR	Days to 50% Flowering	Panicle compactness	Grain Color	Accession Identifier given under the project
14	Leha jowar	Telangana	Adilabad	IS-33649	63	Semi compact	Yellow	
15	Light yellow jowar	Telangana	Khammam	IS-17681	60	Semi compact	Yellow	
16	Madhira Local	Telangana	Khammam	IS-21974	65	Semi compact	Yellow	
17	Madnur	Telangana	Nizamabad	IS-5271	62	Semi compact	Yellow	
18	Menti jonnalu	Telangana	Adilabad	IS-33696	70	Compact	Yellow	
19	Mudda jonna	Telangana	Warangal	IS-36088	70	Compact	Yellow	
20	Mudda patcha jonna	Andhra Pradesh	Kadapa	IS-5106	68	Compact	Yellow	
21	Mudda kanki Jonna	Andhra Pradesh	Ananthapur	IS-37329	66	Compact	Yellow	
22	Mungari patcha jonna	Andhra Pradesh	Nandyal	IS-1141	70	Semi compact	Yellow	
23	Nizam jonna	Andhra Pradesh	East Godavari	IS-5164	62	Semi compact	Yellow	
24	Palam jonna	Telangana	Adilabad	IS-33658	64	Compact	Yellow	
25	Pasara jonna	Andhra Pradesh	Ananthapur	IS-22055	69	Semi compact	Yellow	
26	Patcha jonna	Andhra Pradesh	East Godavari	IS-1422	66	Semi compact	Yellow	
27	Pedda patcha jonna	Andhra Pradesh	Nellore	IS-17789	60	Semi compact	Yellow	PSLRC 4
28	Punasa patcha jonna	Andhra Pradesh	East Godavari	IS-5163	72	Semi compact	Yellow	

S. No.	Local name	State	Location/ District	Accession Identifier given by ICAR-NBPGR	Days to 50% Flowering	Panicle compactness	Grain Color	Accession Identifier given under the project
29	Pyru patcha jonna	Andhra Pradesh	Prakasam	IS-17796	70	Compact	Yellow	PSLRC 6
30	Rangampet jonna	Telangana	Medak	IS-5276	64	Semi compact	Yellow	PSLRC 13
31	Tekedari jonna	Telangana	Adilabad	IS-33623	66	Compact	Yellow	PSLRC 30
32	Vanakalam jola	Andhra Pradesh	Kurnool	IS-22038	67	Semi compact	Yellow	
33	Yellow cholam	Andhra Pradesh	Kurnool	IS-5068	60	Compact	Yellow	PSLRC 12
34	Yellow jowar	Telangana	Khammam	IS-17660	58	Semi compact	Yellow	PSLRC 28
35	Argadi jola	Karnataka	Bidar	IS-5583	58	Compact	Yellow	PSLRC 32
36	Bhagwathi jola	Karnataka	Bijapur	IS-5639	65	Compact	Yellow	PSLRC 16
37	Bhangar kaddi	Karnataka	Raichur	IS-5552	62	Compact	Yellow	PSLRC 34
38	Patch jonna	Karnataka	Bidar	IS-22185	68	Semi compact	Yellow	PSLRC 33
39	Yellow chickni Khajijot	Maharashtra	E.Khandesh	IS-4922	65	Semi compact	Yellow	PSLRC 18
40	Yellow chikni adavad	Maharashtra	E. Khandesh	IS-4917	62	Semi compact	Yellow	
41	Yellow wani	Maharashtra	Akola	IS-17994	60	Semi compact	Yellow	PSLRC 26
42	Paseri jowar	Maharashtra	Satara	IS-40871	62	Compact	Yellow	
43	Peeli Maharashtra	Maharashtra		IS-24366	65	Compact	Yellow	PSLRC 19

S. No.	Local name	State	Location/ District	Accession Identifier given by ICAR-NBPGR	Days to 50% Flowering	Panicle compactness	Grain Color	Accession Identifier given under the project
44	Peeli jowar	Maharashtra	Solapur	IS-22183	60	Semi compact	Yellow	
45	Peeli kalgondi	Maharashtra	Nanded	IS-4606	66	Semi compact	Yellow	PSLRC 35
46	Peeli tarangri	Maharashtra	Parbhani	IS-4568	60	Semi compact	Yellow	PSLRC 36
47	Barari sectagundi	Telangana	Adilabad	IS-5254	65	Loose	Brown	
48	Buramulu jonna	Telangana	Adilabad	IS-33685	69	Loose	Brown	
49	Dekaram jonna	Telangana	Warangal	IS-5225	60	Loose	Brown	
50	Gingri jowar	Telangana	Adilabad	IS-33665	62	Semi compact	Brown	PSLRC 8
51	Jhipri jonna	Telangana	Adilabad	IS-33703	60	Compact	Brown	
52	Jola jonna	Telangana	Nizamabad	IS-5262	64	Compact	Brown	
53	Pedda jonna	Andhra Pradesh	Prakasam	IS-17793	69	Compact	Brown	
54	Pyru yerra jonna	Andhra Pradesh	West Godavari	IS-5152	68	Compact	Brown	PSLRC 9
55	Red jowar	Telangana	Khammam	IS-17687	60	Compact	Brown	PSLRC 10
56	Verra jonna	Andhra Pradesh	Rayachoti	IS-1479	62	Compact	Brown	
57	Wani jowar	Telangana	Adilabad	IS-33648	65	Compact	Brown	
58	Yerra jonna	Andhra Pradesh	Kadapa	IS-17800	60	Compact	Brown	PSLRC 15
59	Yerra jonnalu	Andhra Pradesh	Kurnool	IS-5071	66	Compact	Brown	

S. No.	Local name	State	Location/ District	Accession Identifier given by ICAR-NBPGR	Days to 50% Flowering	Panicle compactness	Grain Color	Accession Identifier given under the project
60	Yerra malle jonna	Andhra Pradesh	Adilabad	IS-33636	70	Semi compact	Brown	PSLRC 11
61	Yerra jonna	Telangana	Karimnagar	IS-5237	70	Semi compact	Brown	
62	Pandhari jowar	Maharashtra	Sholapur	IS-33761	65	Compact	Brown	PSLRC 17
63	Pandhrapur Jowar	Maharashtra	Wadnerganghai	IS-24330	62	Compact	Brown	
64	Lal gunja	Maharashtra	Buldana	IS-4977	72	Compact	Brown	PSLRC 23
65	Lal jowar	Maharashtra	Solapur	IS-33760	70	Compact	Brown	PSLRC 24
66	Lal shallu	Maharashtra	Aurangabad	IS-40738	77	Compact	Brown	PSLRC 25
67	Lalbhindi kolumbi	Maharashtra	Nanded	IS-4599	72	Compact	Brown	PSLRC 27
68	Lalburi	Maharashtra	Dhule	IS-40707	70	Compact	Brown	
69	Lalgunja chirode	Maharashtra	Amaravati	IS-5008	72	Compact	Brown	PSLRC 31
70	Lalgunja chirode	Maharashtra	Amaravati	IS-5008	69	Compact	Brown	
71	Badi bagampath	Telangana	Nizamabad	IS-5269	71	Semi compact	White	
72	Chiruthalavalu	Telangana	Adilabad	IS-33657	65	Semi compact	White	
73	Desavali jonna	Andhra Pradesh	Ananthapur	IS-37330	62	Semi compact	White	
74	Gowrani	Telangana	Adilabad	IS-33620	65	Semi compact	White	
75	Hulgakafi maldandi	Telangana	Adilabad	IS-4578	62	Compact	White	

S. No.	Local name	State	Location/ District	Accession Identifier given by ICAR-NBPGR	Days to 50% Flowering	Panicle compactness	Grain Color	Accession Identifier given under the project
76	Ishimurta tahoor jonna	Telangana	Nizamabad	IS-5259	60	Semi compact	White	
77	Jonna pagadola	Andhra Pradesh	Kurnool	IS-5074	66	Semi compact	White	
78	Juta jonna	Telangana	Adilabad	IS-5246	60	Compact	White	
79	Kobbari jonna	Telangana	Adilabad	IS-33684	60	Compact	White	
80	Konda jonna	Andhra Pradesh	West Godavari	IS-1187	72	Compact	White	
81	Magi jonna	Telangana	Warangal	IS-5216	73	Compact	White	
82	Malli jonna	Telangana	Karimnagar	IS-5233	74	Semi compact	White	
83	Moti	Telangana	Khammam	IS-17725	68	Loose	White	
84	Raichur jonna	Andhra Pradesh	Kurnool	IS-34003	66	Semi compact	White	
85	Ratnapure jonna	Telangana	Karimnagar	IS-5234	62	Compact	White	
86	Rayachur jonna	Telangana	Mahaboobnagar	IS-17726	65	Semi compact	White	
87	Sai jonna	Telangana	Adilabad	IS-33686	65	Semi compact	White	PSLRC 3
88	Shivuni thalavalu	Telangana	Adilabad	IS-33693	62	Semi compact	White	
89	Tandur Local	Telangana	Ranga Reddy	IS-36451	60	Semi compact	White	PSLRC 29
90	Thithri jonna	Telangana	Adilabad	IS-33711	60	Loose	White	

S. No.	Local name	State	Location/ District	Accession Identifier given by ICAR-NBPGR	Days to 50% Flowering	Panicle compactness	Grain Color	Accession Identifier given under the project
91	Yendakalam jonna	Telangana	Mahabubnagar	IS-40335	65	Compact	White	
92	Allu jonna	Karnataka	Raichur	IS-5566	68	Semi compact	White	
93	Ankulga	Karnataka	Gulbarga	IS-33887	62	Compact	White	
94	Badi jowar	Karnataka	Bidar	IS-37183	60	Semi compact	White	
95	Benni jola	Karnataka	Bellary	IS-37311	62	Semi compact	White	
96	Besigai jola	Karnataka	Chikmaglur	IS-5480	60	Semi compact	White	
97	Raichur jola	Karnataka	Bellary	IS-22088	65	Compact	White	
98	Raichur jonna	Karnataka	Bellary	IS-22085	64	Semi compact	White	
99	Raichur Jowar	Karnataka	Raichur	IS-22105	62	Semi compact	White	
100	Kaki jonna	Telangana		IS-22197	66	Semi compact	White black glume	PSLRC 22
101	Kakimuttani jonna	Telangana	Medak	IS-5285	68	Semi compact	White black glume	
102	Kakivai jonna	Andhra Pradesh	Nellore	IS-17758	69	Semi compact	White black glume	
103	Nila jonna	Telangana	Karimnagar	IS-5238	66	Loose	White black glume	PSLRC 21
104	Pandori wani	Maharashtra	Akola	IS-18018	65	Semi compact	White black glume	
105	Pandri	Maharashtra	Osmanabad	IS-4573	66	Semi compact	White black glume	
106	Pandri garva datoda	Maharashtra	Buldana	IS-4957	66	Semi Compact	White black glume	

S. No.	Local name	State	Location/ District	Accession Identifier given by ICAR-NBPGR	Days to 50% Flowering	Panicle compactness	Grain Color	Accession Identifier given under the project
107	Pandri halki chirode	Maharashtra	Amaravati	IS-5006	62	Semi compact	White black glume	
108	Pandri jowar	Maharashtra	Parbhani	IS-40784	68	Semi compact	White black glume	PSLRC 20

SUPPLEMENTARY TABLE S2: Analysis of variance for grain mold disease reaction of sorghum genotypes

**Source of variation**	**df**	**Sum of squares**	**Mean Squares**	**F ratio**	**Probability**
Environments	2	0.03079	0.015	0.051	0.950
Treatments	20	246.92	12.346	40.849	0.000***
Pooled error	120	47.73	0.397		
Joint pooled error	160	48.35	0.302		

**Comparisons**	**S. Error**	**S.E.Diff.**	**C.D. 5%**
Treatments	0.183	0.259	0.511
Environments	0.069	0.098	0.193
Treatments x Env	0.317	0.448	0.886

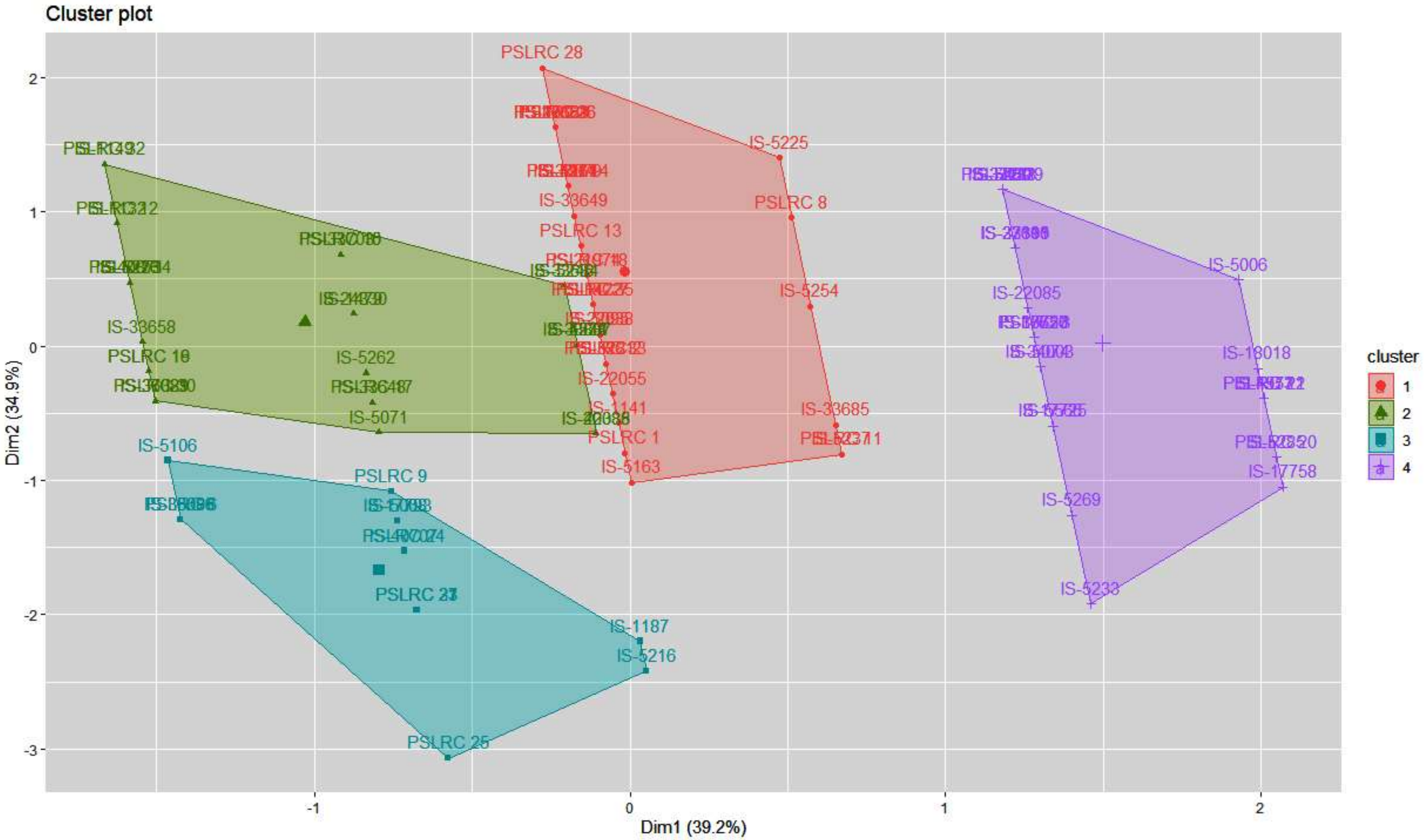

Supplementary Figure 1 Cluster/PCA plot showing diversity of 108 sorghum landraces for the analyzed morphological traits

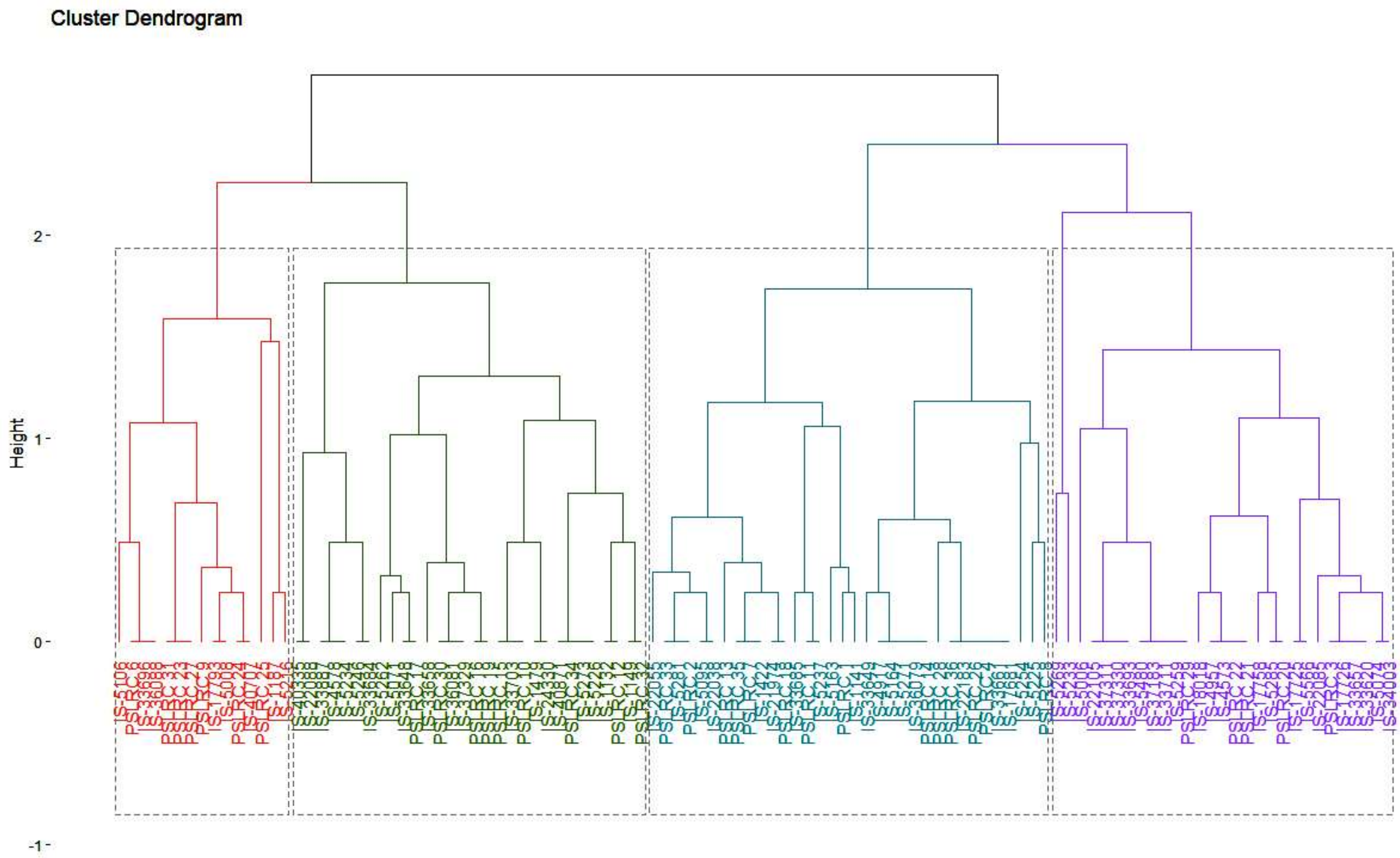

Supplementary Figure 2: Dendrogram of 108 sorghum landraces for morphological traits based on growth distance matrix

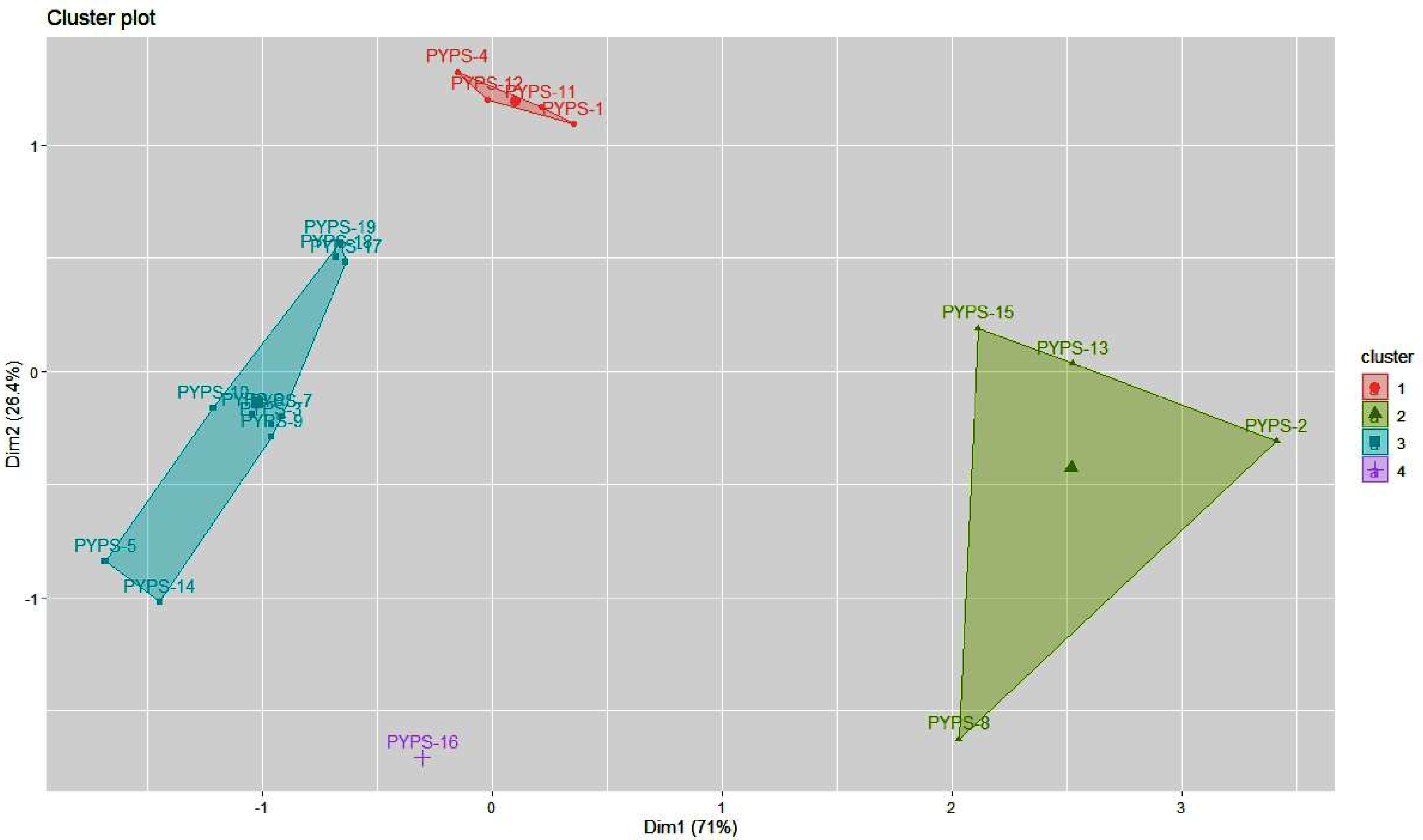

Supplementary Figure 3: Cluster/PCA plot demonstrating diversity of 19 sorghum varieties developed from landraces

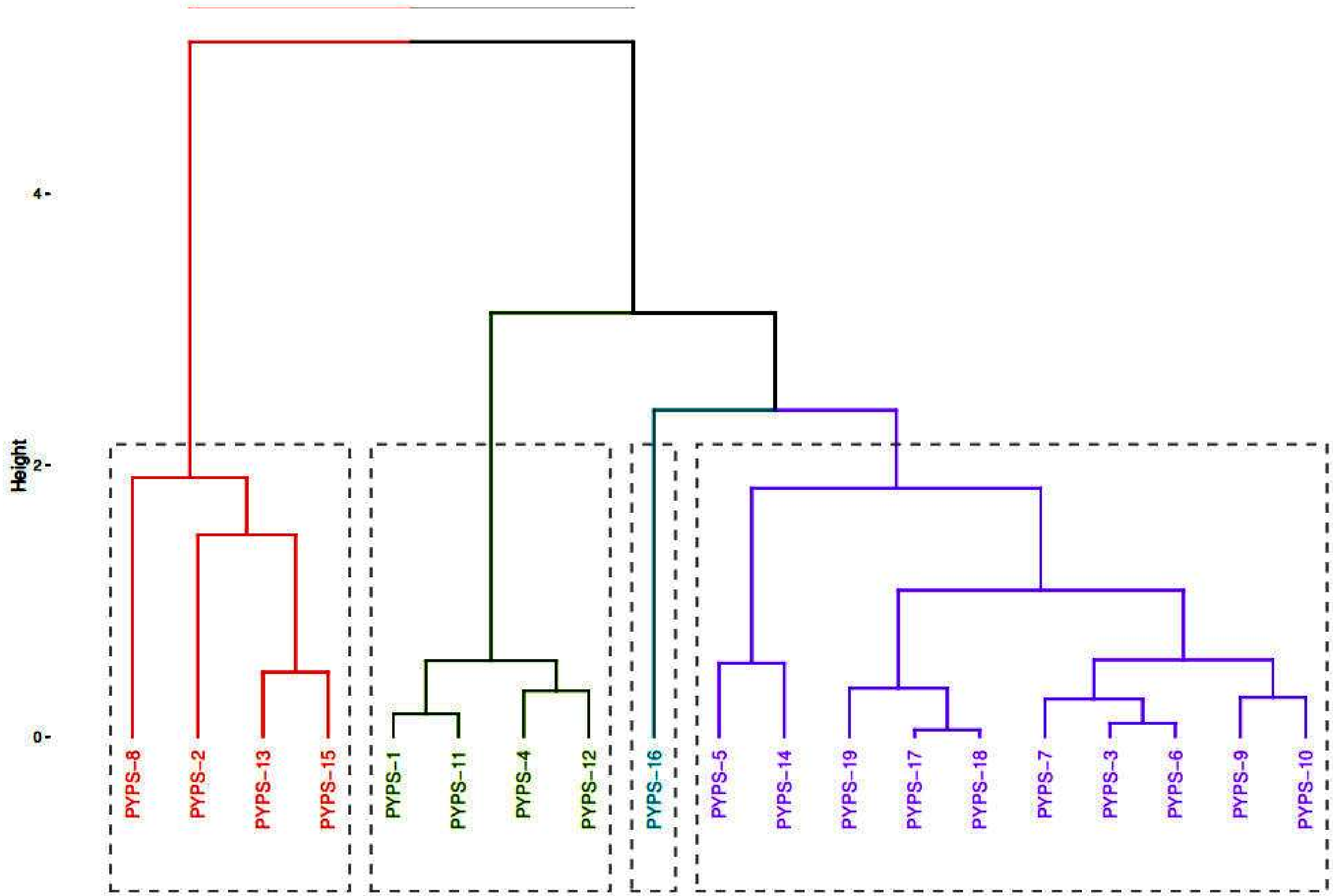

Supplementary Figure 4: Dendrogram of 19 sorghum varieties developed from landraces

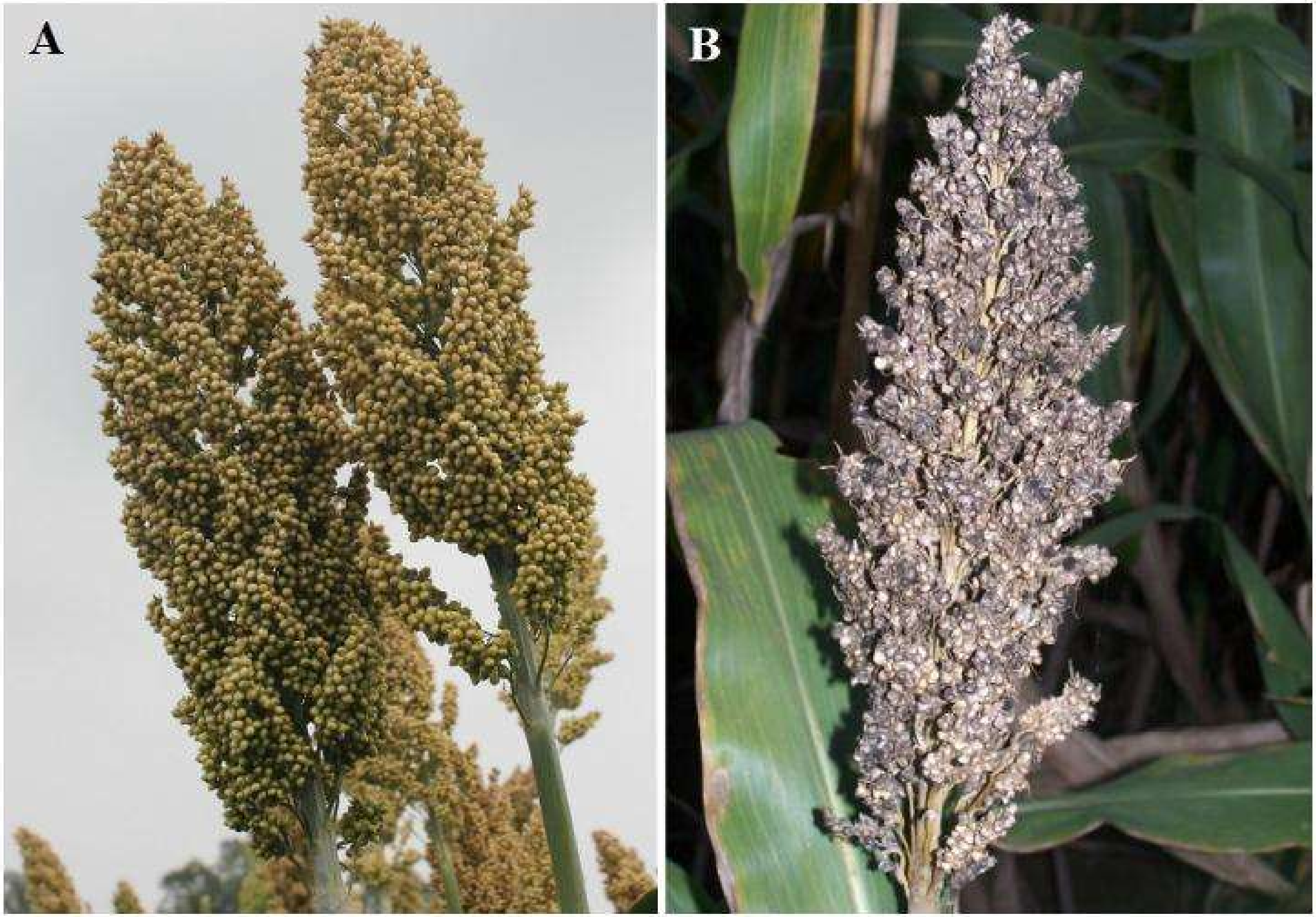

Supplementary Figure 5: A. Sorghum panicles free from disease and B. Grain mold affected sorghum panicles
